# Landscape genetics reveals unique and shared effects of urbanization for two sympatric pool‐breeding amphibians

**DOI:** 10.1002/ece3.5685

**Published:** 2019-10-01

**Authors:** Jared J. Homola, Cynthia S. Loftin, Michael T. Kinnison

**Affiliations:** ^1^ School of Biology and Ecology University of Maine Orono ME USA; ^2^ Maine Cooperative Fish and Wildlife Research Unit U.S. Geological Survey Orono ME USA

**Keywords:** circuit theory, landscape genetics, microsatellites, urbanization, vernal pool

## Abstract

Metapopulation‐structured species can be negatively affected when landscape fragmentation impairs connectivity. We investigated the effects of urbanization on genetic diversity and gene flow for two sympatric amphibian species, spotted salamanders (*Ambystoma maculatum*) and wood frogs (*Lithobates sylvaticus*), across a large (>35,000 km^2^) landscape in Maine, USA, containing numerous natural and anthropogenic gradients. Isolation‐by‐distance (IBD) patterns differed between the species. Spotted salamanders showed a linear and relatively high variance relationship between genetic and geographic distances (*r* = .057, *p* < .001), whereas wood frogs exhibited a strongly nonlinear and lower variance relationship (*r* = 0.429, *p* < .001). Scale dependence analysis of IBD found gene flow has its most predictable influence (strongest IBD correlations) at distances up to 9 km for spotted salamanders and up to 6 km for wood frogs. Estimated effective migration surfaces revealed contrasting patterns of high and low genetic diversity and gene flow between the two species. Population isolation, quantified as the mean IBD residuals for each population, was associated with local urbanization and less genetic diversity in both species. The influence of geographic proximity and urbanization on population connectivity was further supported by distance‐based redundancy analysis and multiple matrix regression with randomization. Resistance surface modeling found interpopulation connectivity to be influenced by developed land cover, light roads, interstates, and topography for both species, plus secondary roads and rivers for wood frogs. Our results highlight the influence of anthropogenic landscape features within the context of natural features and broad spatial genetic patterns, in turn supporting the premise that while urbanization significantly restricts interpopulation connectivity for wood frogs and spotted salamanders, specific landscape elements have unique effects on these two sympatric species.

## Graphical abstract

Landscape genetic analyses reveal contrasting patterns of genetic diversity and differentiation for wood frogs and spotted salamanders across an overlapping region of their ranges that includes numerous natural and anthropogenic gradients. Urban landscape elements have detectable, but differing effects on each species. The scale dependence of isolation‐by‐distance (IBD) patterns is empirically investigated, revealing how IBD inferences vary depending on the spatial extent considered.

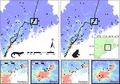

## 1 INTRODUCTION

Landscape alterations that accompany increases in human population density (i.e., urbanization) commonly influence ecological and evolutionary processes. For instance, landscape fragmentation and habitat loss can lead to reduced connectivity among wildlife populations, consequently disrupting demographic support from metapopulation dynamics that would otherwise improve population stability via immigration (Andrén, 1994; Crosby, Licht, & Fu, 2008). Reductions in connectivity are potentially associated with reduced levels of gene flow and genetic diversity (Crawford, Peterman, Kuhns, & Eggert, 2016; Ortego, Aguirre, Noguerales, & Cordero, 2015) and an increased risk of extirpation from lost demographic support (Bascompte & Sole, 1996; Haddad et al., 2015; Wilcox & Murphy, 1985). Populations that do persist may be subject to decreased fitness associated with inbreeding depression (Andersen, Fog, & Damgaard, 2004; Lopez, Rousset, Shaw, Shaw, & Ronce, 2009). These issues are of immediate concern as urban areas are becoming larger and more prevalent worldwide. For instance, growth in global human population size (UNDESA, 2012, 2015) has been accompanied by increases in the percentage of people living in urban areas from 29.4% in 1950 to 52.1% in 2011 and up to 70% anticipated by 2050 (UNDESA, 2012). During the past century, cities have also become increasingly diffuse, leading to a greater proportion of the landscape being affected by their growth (Seto, Sánchez‐Rodríguez, & Fragkias, 2010; Theobald, 2010). Given these trends, the ecological and evolutionary effects of urbanization on wildlife are likely to intensify in the coming decades.

Metapopulation‐structured species are especially vulnerable to negative effects of habitat fragmentation associated with urbanization (Graham, Haines‐Young, & Field, 2017). These species are spatially arranged in discrete subpopulations that are spread across a heterogeneous landscape and are dependent to a degree on dispersal among constituent subpopulations (Hanski, 1998). In classical metapopulation theory, subpopulations experience bouts of extinction and recolonization while maintaining overall metapopulation stability (Hanski, 1991; Levins, 1969). A loss of connectivity among subpopulations has been predicted to result in local extirpation and abundance declines for metapopulations (Grilli, Barabás, & Allesina, 2015; Reigada, Schreiber, Altermatt, & Holyoak, 2015; Schnell, Harris, Pimm, & Russell, 2013), a result that has been observed in several natural systems (Crooks et al., 2017). In some cases, habitat fragmentation has allowed evolutionary forces to generate changes in phenotypic traits, influencing characteristics such as dispersal propensities (Cheptou, Hargreaves, Bonte, & Jacquemyn, 2017) or life history traits (De Roissart, Wybouw, Renault, Leeuwen, & Bonte, 2016), as well as broader eco‐evolutionary processes (Fronhofer & Altermatt, 2017). The metapopulation concept has proven broadly applicable in urbanization‐associated habitat fragmentation scenarios, facilitating an improved understanding of how landscape alterations can affect regional population processes for many taxa, such as birds (Millsap, 2018; Padilla & Rodewald, 2015) and amphibians (Cox, Maes, Calster, & Mergeay, 2017; Hale et al., 2013; Heard, McCarthy, Scroggie, Baumgartner, & Parris, 2013).

Landscape genetics provides a framework for evaluating support for potential environmental correlates of observed interpopulation structure, allowing for the generation of inferences of spatially explicit drivers of gene flow. This approach has revealed allele frequency changes associated with diminished gene flow due to habitat fragmentation (Epps & Keyghobadi, 2015; Zellmer & Knowles, 2009). Studies focused on disentangling effects of natural versus anthropogenic landscape elements, and using multiple species to obtain a more comprehensive understanding of connectivity, have been highlighted as critical areas for advancing landscape genetics research (Manel & Holderegger, 2013; Richardson, Brady, Wang, & Spear, 2016). However, detecting landscape genetic effects of anthropogenic fragmentation is not without challenges. Unlike many natural landscape features present since deglaciation, the widespread appearance of anthropogenic features has occurred relatively recently, limiting the opportunity for drift or gene flow to affect gene frequencies, and placing constraints on statistical power. This necessitates sampling adequate numbers of populations at geographic scales where disruptions to gene flow are most apt to affect background patterns of drift–migration equilibrium.

Habitat losses, such as the landscape changes coincident with urbanization, have been singled out as a leading threat to amphibian species, with empirical evidence mounting for population declines associated with changes in land cover (Price, Dorcas, Gallant, Klaver, & Willson, 2006), canopy cover (Clark, Reed, Tavernia, Windmiller, & Regosin, 2008), and roadways (Andrews, Gibbons, & Jochimsen, 2008). Ecological studies have consistently suggested that pool‐breeding amphibians may be particularly susceptible to negative effects of landscape fragmentation (Baldwin & de Maynadier, 2009; Semlitsch, 2003). For instance, because they require access to both wetland and upland environments to complete their semiaquatic life cycle (Semlitsch, 2008), any barriers between those two environments could impair a population (Homan, Windmiller, & Reed, 2004). Additionally, the ability to occasionally disperse among populations is important for these metapopulation‐structured amphibian species due to highly variable interannual recruitment success at the local scales of pools (Baldwin, Calhoun, & de Maynadier, 2006b; Green, Hooten, Grant, & Bailey, 2013). Tracking studies suggest altered habitats between pools can reduce overall dispersal propensities (Cline & Hunter, 2014, 2016). Despite these observed ecological effects, signals of urbanization‐related influences on the genetic structure of pool‐breeding amphibians have not been consistently detected (e.g., Coster, Babbitt, Cooper, & Kovach, 2015; Peterman et al., 2015; Richardson, 2012; Table 1). In some of these cases, a lack of effect could be associated with the surveyed spatial extents that were relatively small and that may not be commensurate with the scales over which drift–migration equilibrium is most disrupted and detectable.

**Table 1:** Studies of the effects of urban landscape elements (e.g., roads and developed lands) on connectivity of pool‐breeding amphibians based on microsatellite genotyping. Negative (↘) and negligible effects (↔) are noted

Species	Approx. study area (km^2^)	No. populations	Effect	Citation
Blanchard's cricket frogs (*Acris blanchardi*)	2,320	28	↘	Youngquist, Inoue,Berg, and Boone (2017)
Columbia spotted frogs (*Rana luteiventris*)	213	8	↘	Goldberg and Waits (2010)
Eastern tiger salamander (*Ambystoma tigrinum*)	220	26	↔	Titus, Bell, Becker, and Zamudio (2014)
Long‐toed salamanders (*Ambystoma macrodactylum*)	213	4	↘	Goldberg and Waits (2010)
Natterjack toad (*Epidalea calamita*)	40	23	↘	Cox et al. (2017)
Ringed salamander (*Ambystoma annulatum*)	35	20	↔	Peterman et al. (2015)
Spotted salamander (*Ambystoma maculatum*)	35	23	↔	Peterman et al. (2015)
Spotted salamander (*Ambystoma maculatum*)	2,080	23	↘	Coster, Babbitt, Cooper, et al. (2015)
Spotted salamander (*Ambystoma maculatum*)	21,000	22	↘	Richardson (2012)
Wood frog (*Lithobates sylvaticus*)	200	9	↔	Peterman et al. (2013)
Wood frog (*Lithobates sylvaticus*)	375	65	↔	Gabrielsen, Kovach, Babbitt, and McDowell (2013)
Wood frog (*Lithobates sylvaticus*)	2,080	20	↔	Coster, Babbitt, Cooper, et al. (2015)
Wood frog (*Lithobates sylvaticus*)	21,000	22	↘	Richardson (2012)

We investigated the effects of urbanization on metapopulation processes by examining the landscape genetics of two metapopulation‐structured pool‐breeding amphibians, spotted salamanders (*Ambystoma maculatum*) and wood frogs (*Lithobates sylvaticus*), in an area of overlap between their native ranges. Several characteristics make these species excellent subjects for evaluating the effects of urbanization with a landscape genetics approach. For instance, the species have small annual home ranges (spotted salamanders: up to 301 m^2^, Ousterhout and Burkhart, 2017; wood frogs: up to 32,165 m^2^, Blomquist & Hunter, 2010; Groff, Calhoun & Loftin, 2017), relatively short generation times (spotted salamanders: maturity in 2–7 years, Flageole & Leclair, 1992; wood frogs: maturity in 2–3 years, Sagor, Ouellet, Barten, & Green, 1998), and high rates of philopatry (Vasconcelos & Calhoun, 2004). Both species are vulnerable to degradation of the breeding sites they share within their overlapping ranges in the northeastern United States (Harper, Rittenhouse, & Semlitsch, 2008). These two amphibians have several differentiating life history and behavioral attributes that likely affect how urbanization influences their interpopulation dynamics. For instance, wood frogs tend to be shorter lived, have larger home ranges, and are more vagile than spotted salamanders (Berven & Grudzien, 1990; Madison, 1997; Semlitsch, 1998). Due to these characteristics, we expected the magnitude and dynamics of urbanization‐related effects to differ between the species. We tested three sets of hypotheses to examine effects of urbanization on individual populations, interpopulation dynamics, and genetic structure more broadly.

1. *Broadscale patterns of genetic structure*: We hypothesized broadscale patterns of genetic structure will be much stronger for wood frogs than spotted salamanders. Previous work to characterize the isolation‐by‐distance (IBD) relationships for these two species has supported this hypothesis through observations of clear positive correlations between geographic and genetic distances for wood frogs (Crosby et al., 2008; Peterman, Feist, Semlitsch, & Eggert, 2013; Richardson, 2012; Squire & Newman, 2002) and either high variance positive correlations (Burkhart et al., 2017; Peterman et al., 2015; Richardson, 2012; Zamudio & Wieczorek, 2007) or nonsignificant relationships (Purrenhage, Niewiarowski, & Moore, 2009; Whiteley, McGarigal, & Schwartz, 2014) for spotted salamanders. Additionally, we hypothesize that IBD relationships within species are not absolute, but are instead scale‐dependent, such that the strongest correlations between genetic isolation and distance will occur at some intermediate geographic scales of analysis. Below these scales, the strength of IBD is expected to be weaker due to the highly variable patterns of local dispersal and gene flow overwhelming drift, and limited sample size. Above these scales, sample sizes are greater, but drift–migration equilibrium may take longer to occur, and stochastic processes weaken correlations. Understanding the scale dependence of IBD may be important to understanding why different studies on a specific species obtain different IBD inferences and help to inform whether an IBD study has sufficient power to identify effects of landscape features on population connectivity.
2. *Urbanization's influence on isolation and genetic diversity*: We hypothesized that greater urbanization will affect metapopulation dynamics by increasing among‐population isolation and reducing within‐population genetic diversity for both species, relative to patterns in less‐urbanized areas (Frankham, 2015; Kenney, Allendorf, Mcdougal, & Smith, 2014; Pavlova et al., 2017). We further hypothesize that the substantive differences in life history and vagility of our focal species will contribute to different effects of the same landscape features (Moyle, 2006; Phillipsen et al., 2015). Wood frogs are often more mobile than spotted salamanders and may be more capable of traversing urbanization features than salamanders. However, this increased mobility may increase wood frog exposure to deleterious features of the broader urbanized landscape relative to spotted salamanders.
3. *Landscape features influencing gene flow*: We predict signals of diminished gene flow associated with anthropogenic landscape features will be present; however, they will be weaker than signals from natural features (e.g., rivers, elevation gradients). Landguth et al. (2010) used simulations to illustrate a lag time of approximately 200 generations when using a landscape genetics approach to detect signals of barriers, although they note the magnitude of this effect likely varies with each population's effective size. The influence of lag time is also supported by empirical research that considers effects of both natural and anthropogenic features on amphibian gene flow (Garcia, Ivy, & Fu, 2017; Peterman et al., 2015; Richardson, 2012). Due to this lag, most natural features will likely have stronger signals, such as rivers appearing more important than interstate highways. However, given the magnitude of expected effects for heavily urbanized areas, we anticipate that the landscape features that coincide with the most intense urbanization (e.g., road density) will be the strongest predictors of isolation.

## 2 MATERIALS AND METHODS

### 2.1 Sample collection

**Figure 1:**
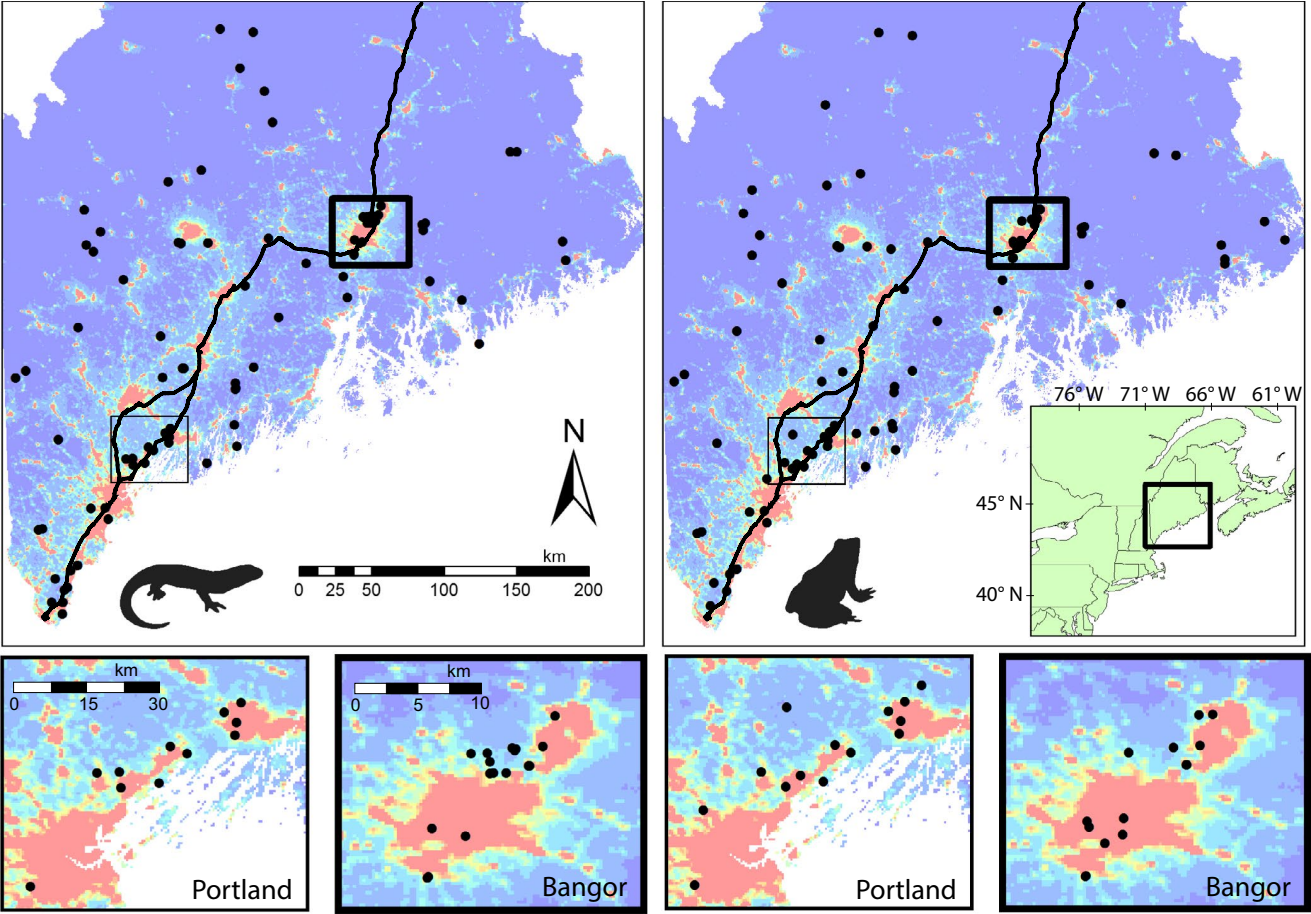
Locations of 90 wood frog and 87 spotted salamander vernal pool sampling sites in Maine, USA. Inset maps illustrate densely sampled regions around Portland (thin outline) and Bangor, Maine (heavy outline). Solid black line in largest extent maps represents interstate highways. Red background coloration indicates high levels of nighttime light intensity based on NASA Visible Infrared Imaging Radiometer Suite (VIIRS) data and is provided as a proxy for human population density

Larval and embryonic wood frogs and spotted salamanders were collected from vernal pools throughout Maine, USA (Figure 1). We sampled across a 35,000‐km^2^ region that contains several natural (e.g., topographic) and anthropogenic (e.g., urbanization) landscape gradients. Sampling effort was concentrated in areas of urbanization to ensure adequate power to detect effects of relatively recent urban‐associated landscape features (Balkenhol, Cushman, Waits, & Storfer, 2016) with additional sampling conducted away in rural areas to facilitate appropriate comparisons to natural landscape features. When a site (treated throughout the study as a population) was visited prior to egg hatching, we sampled up to 40 egg masses, collecting one embryonic individual from each mass to reduce the likelihood of sampling siblings because the inclusion of closely related individuals has been found to bias some genetic analyses (Goldberg & Waits, 2010, Rodríguez‐Ramilo & Wang, 2012, Peterman, Brocato, Semlitsch, & Eggert, 2016, Wang, 2018, but see Waples and Anderson (2017)). If larvae were free‐swimming upon sampling, a small dipnet was used to collect individuals from throughout the pool and full siblings were later removed based on sibship analyses (see below). Sampling occurred during April and May 2014, 2015, and 2016. When fewer than 25 individuals were collected at a site in one year, we returned to sample in the subsequent year.

### 2.2 Genetic data collection and quality control

Genomic DNA was extracted from whole embryonic or larval individuals using Qiagen DNeasy kits following the manufacturer's instructions. We analyzed variability at 10 microsatellite loci to evaluate spatial genetic structure for each species. PCR components, thermal cycler profiles, and citations for loci primer sequences are described in Appendix S1. Negative controls were included in each 96‐well PCR to allow for detection of reagent contamination. Microsatellite fragment analysis was conducted using an ABI 3730 automated genetic analyzer (Applied Biosystems, Inc.) at the University of Maine DNA Sequencing Facility. Genotyping was performed using Geneious v7.1.9 with fragment sizes based on GeneScan 500 LIZ Size Standard (Applied Biosystems) and all allele calls confirmed visually. A random 10% of individuals were genotyped a second time to evaluate genotype error rates.

A series of data filtering steps was performed to reduce the potential influence of sampling bias and to ensure conformance to assumptions of population genetic analyses. First, individuals with fewer than five successfully amplified loci were removed. Peterman et al. (2016) found five microsatellite loci to be as informative as both 10 and 15 loci for estimating heterozygosity and allelic richness in other spotted salamander populations. Next, to reduce the likelihood of mischaracterizing allele frequencies due to small sample sizes, we eliminated sites with fewer than ten individuals successfully genotyped. Finally, we performed sibship reconstruction for all individuals sampled at each site using COLONY (v2.0.5.9; Jones & Wang, 2010; Wang, 2004) and subsequently haphazardly removed all but one individual from any apparent full‐sibling family. COLONY analyses assumed polygamy in both sexes, no inbreeding, and were performed using a long run with the full likelihood method. In addition to minimizing the degree of family structure present in our sample set, this post hoc removal of siblings improves congruence in sampling design between populations sampled at the egg stage and those sampled as free‐swimming larvae where inadvertently collecting siblings is more likely.

We estimated the frequency of null alleles for each locus and tested for Hardy–Weinberg equilibrium for each locus–sampling site combination using PopGenReport (Adamack & Gruber, 2014) in R v3.4.1 (R Core Team, 2016). Independent sorting of genotypes (i.e., linkage disequilibrium) was evaluated using exact testing in Arlequin v3.5.1.2 (Excoffier & Lischer, 2010). Alpha levels to determine statistical significance for tests of Hardy–Weinberg proportions and independent sorting of genotypes were adjusted using the false discovery rate (FDR) approach of Benjamini and Hochberg (1995) based upon a 0.05 alpha level.

### 2.3 Genetic diversity and differentiation

We quantified genetic diversity within each site and genetic differentiation among sites using multiple measures. Average number of alleles per site (A_O_) was estimated using PopGenReport, and allelic richness (i.e., allelic counts rarefied based on smallest sample size per species; spotted salamander: 10, wood frog: 12; AR), expected heterozygosity (*H*_E_), and Wright's inbreeding coefficient (*F*_IS_) were estimated using the R package hierfstat (Goudet & Jombart, 2015). Genetic differentiation was calculated using *G*_ST_ (i.e., Nei, 1973; Nei & Chesser, 1983) and *G″*_ST_ (Meirmans & Hedrick, 2011). *G*_ST_ (commonly reported as *F*_ST_) summarizes the amount of diversity contained among populations relative to the diversity of all populations combined (Nei, 1973), whereas *G″*_ST_ provides a scaled maximum value of *G*_ST_ based on the genetic diversity within a measured population (Meirmans & Hedrick, 2011). Both *G*_ST_ and *G″*_ST_ were estimated using the R package mmod (Winter, 2012). Statistical significance of pairwise population differentiation was evaluated with an exact G test implemented using the genetic differentiation option in Genepop v4.2 (Raymond & Rousset, 1995; Rousset, 2008) with a FDR correction for type I error rates.

The spatial arrangement of effective genetic diversity was visualized using estimated effective migration surfaces (EEMS; Petkova, Novembre, & Stephens, 2016). Effective genetic diversity reflects the expected genetic dissimilarity of two individuals sampled within each deme assuming a generally IBD‐driven system and a stepping‐stone dispersal pattern (Petkova et al., 2016). EEMS constructs a dense, regular grid across the study range and assigns sampling sites to the nearest grid intersection (node), often resulting in a set of fewer demes than the actual number of sampling sites. Diversity values are then interpolated among the demes to create a continuous surface for visualizing spatial patterns. Our starting grid provided 500 potential nodes for deme assignment, of which 462 were incorporated into the analysis due to the irregular landscape boundaries. Previous work has demonstrated EEMS results to be qualitatively robust when various numbers of nodes were used in analyses (Petkova et al., 2016). EEMS analysis parameters were adjusted to achieve the recommended 20%–40% acceptance rates before running the analysis using 1 × 10^7^ iterations, a burn‐in period of 1 × 10^6^ iterations, and a thinning interval of 1 × 10^3^ (Combs, Puckett, Richardson, Mims, & Munshi‐South, 2017; Petkova et al., 2016). All EEMS plotting was performed using rEEMSplots R package (Petkova et al., 2016).

We also evaluated all populations for the presence of bottlenecks that may be associated with urbanization using the program Bottleneck (Cornuet & Luikart, 1996; Piry, Luikart, & Cornuet, 1999). We used the two‐phase model of microsatellite mutation (TPM; Di Rienzo et al., 1994) with variance set to 12 and the probability of single‐step mutations set to 95% as recommended by Piry et al. (1999). Significance was evaluated using a one‐tail Wilcoxon test with an FDR‐adjusted alpha level.

### 2.4 Isolation by distance

To examine IBD relationships, we compared each pairwise measure of genetic differentiation with between‐site Euclidean geographic distance. Genetic differentiation measures were linearized (*G*_ST_/(1 − *G*_ST_)) as suggested by Slatkin (1995), and geographic distances were measured as straight‐line Euclidean distances using the “distm” function of the R package geosphere (Hijmans, 2017). We examined relationships between linearized *G*_ST_ and both log‐transformed and nontransformed geographic distances. The slope of IBD relationships based on log transformation of geographic distance is useful for understanding the dispersal kernel relationships in scenarios of two‐dimensional movements (Rousset, 2000), whereas nontransformed distances are helpful for understanding broadscale patterns of IBD (sensu Hutchison & Templeton, 1999). While we provide the slope of the IBD relationship based on log‐transformed geographic distances, it is important to note that our study design does not meet the assumptions for actually estimating dispersal kernel size per se (e.g., sampling extent greater than 0.56*σ*/√2*μ*, where *σ* is the parent–offspring axial distance and *μ* is the mutation rate of the loci; Rousset, 2004), and key parameters are unknown (i.e., *D*, the effective density). Therefore, the regression slopes we report should be considered a broad approximation of observed increases in genetic differentiation with geographic distance (*Dσ* ^2^) and useful only for comparisons between species within this specific study. Associations between the distance matrices were tested using regression and Mantel tests (Mantel, 1967) that were implemented in the R package vegan (Oksanen et al., 2017). We evaluated Mantel tests for significance based on 9,999 permutations.

We examined the relationship between genetic and geographic distances as a function of the spatial scale of analysis using two methods. First, we constructed a Mantel correlogram (Borcard & Legendre, 2012; Legendre & Legendre, 2012; Oden & Sokal, 1986) to quantify the strength of the relationship between genetic and geographic distances within various distance classes using the “mantel.correlog” function in vegan. Distance class breakpoints were placed every 20 km, and larger distance classes that did not contain every sampling site were omitted to avoid bias (Wagner et al., 2005). Statistical significance of correlations was assessed using 10,000 permutations and a FDR correction based on a 0.05 alpha level. Next, we estimated the slope (*β*) of variable intercept IBD regressions that were performed repeatedly using expanding datasets based on distance between sampling sites, which generated IBD scaling profiles for each species. For example, the first iteration of the analysis was conducted using the 20 shortest pairwise geographic distances, the next iteration with the 21 shortest, and so on, until all pairwise comparisons were included. We performed 1,000 bootstrapped replicates of each regression using the “Boot” function in the R package car (Fox & Weisberg, 2011) to estimate each beta coefficient and its 95% confidence intervals. The slope confidence intervals for each IBD regression were then plotted using the maximum analyzed distance as a response variable to generate the IBD scaling profiles.

We also used EEMS to visualize spatial patterns of connectivity among sampling sites. When assessing connectivity, EEMS identifies areas with greater differentiation than expected between neighboring demes assuming a generally IBD‐driven system and a stepping‐stone dispersal pattern (Kimura & Weiss, 1964; Petkova et al., 2016). The number of effective migrants among sites is then interpolated to construct a graphic depiction of connectivity across the landscape.

### 2.5 Regression and multivariate analyses

We assessed the influence of urbanization on a sampling site's degree of isolation and genetic diversity. The intensity of urbanization near a site was quantified using six environmental characteristics measured in ArcGIS v10.2 (ESRI): distance to nearest roadway, percent impervious surface within one km, length of roads within 1 km for light, secondary, and primary road types, and percent canopy cover within 1 km. Road type and classification was determined using the State of Maine's NG911 Roads dataset (http://www.maine.gov/megis/catalog/, accessed Feb 18, 2016); impervious surface extent was based on the Maine Department of Inland Fisheries and Wildlife's July 2016 impervious surface dataset (J. Czapiga, Maine Department of Inland Fisheries and Wildlife, unpublished data); and percent canopy cover data were drawn from the 2011 National Land Cover Database (Homer et al., 2015). Collinearity between the urbanization‐related explanatory variables was evaluated, and one variable was selected at random to be retained from each set with a correlation coefficient exceeding 0.7.

We quantified the degree of isolation experienced at each sampling site by averaging the residuals of the IBD data points that include that site. This approach is similar to the decomposed pairwise regression analysis to detect outlier populations described by Koizumi, Yamamoto, and Maekawa (2006) and essentially provides an index of genetic differentiation corrected for geographic distance. Sites with the largest average residual values were presumed to be more isolated than those with smaller average residuals. Due to strong correlation between *G*_ST_ and *G″*_ST_ for both species (spotted salamander *r* = .993, wood frog *r* = .979), we quantified isolation using only the *G*_ST_‐based IBD relationships. Genetic diversity relationships were assessed using *H*_E_ and AR. We conducted three statistical analyses to test hypotheses concerning the relationship between these three factors: multiple regression between each measure of genetic diversity and all retained urbanization variables, multiple regression between urbanization variables and degree of site isolation, and simple linear regression between each measure of genetic diversity and degree of isolation.

We examined the influence of urbanization and spatial proximity to observed interpopulation genetic differentiation with two approaches complementary to the above IBD and regression analyses: distance‐based redundancy analysis (dbRDA) and multiple matrix regression with randomization (MMRR). We conducted dbRDA using the “capscale” function and examined the significance of individual model terms using 10,000 permutations with the “anova.cca” function in vegan. Our global dbRDA model included pairwise *G*_ST_ values in the response matrix, the above‐described urbanization‐related metrics in an explanatory matrix, and a conditional matrix containing the latitude and longitude of each site in decimal degrees. Model terms were eliminated using a backward optimization procedure where nonsignificant terms were removed, and a simplified model was tested until all remaining terms were significant. MMRR provides a multivariate method for examining the relationships between a response matrix (e.g., interpopulation genetic divergence) and multiple explanatory matrices (e.g., environmental characteristics) while accounting for interpopulation geographic distances (Wang, 2013). With data included in the dbRDA global model, we implemented MMRR using the “lgrMMRR” function in PopGenReport, which involved 10,000 permutations to allow statistical significance to be evaluated based on the pseudo‐*t* statistic of Legendre, Lapointe, and Casgrain (1994).

### 2.6 Landscape resistance modeling

We tested support for a series of resistance surface models to determine the relative influence of ten landscape features on the genetic structuring of each species. The modeling was a two‐step process. First, we optimized resistance values for each feature, and then, we conducted generalized additive modeling to determine which features were most influential for each species. Features to be analyzed were generally selected based on data availability and previous resistance modeling for these species (Richardson, 2012). Land cover was based on the 2011 National Land Cover Database (NLCD; Homer et al., 2015) and merged into three class that generally describe forests (land cover A), open areas and agriculture (land cover B), and developed areas and open waterbodies (land cover C; Table 2; Richardson, 2012). Road data were derived from the State of Maine NG911 Roads dataset and sorted into three classes describing limited‐access interstate freeways, secondary roads (e.g., state highways), and light roads. We subset river data from the National Hydrography Dataset (USGS, https://nhd.usgs.gov/, access Feb. 18, 2016) into two classes based on the Strahler numbering system. Medium rivers included order 4 and 5 streams; large rivers included order 6 and 7 streams; and lower order waterways were not considered. Railway data were based on the Maine Department of Transportation's RailRouteSys dataset (Johnson et al., 2011). We calculated a terrain ruggedness index (TRI; Riley, DeGloria, & Elliot, 1999) using the “tri” function in the R package spatialEco (Evans, 2017) to characterize topographic heterogeneity. Raster processing was performed using ArcGIS v10.2. Processing included buffering all linear features to ensure their continuity following conversion to a raster and the resampling of all rasters to a 90‐m resolution, which was necessary given computation constraints owing to the extent of the landscape being processed.

**Table 2:** Reclassification of National Land Cover Database fields into three categories for use in resistance surface modeling

NLCD category	NLCD descriptions	Assigned category
41	Deciduous forest	A
42	Evergreen forest	A
43	Mixed forest	A
90	Woody wetlands	A
95	Emergent herbaceous wetlands	A
21	Developed, open space	B
52	Shrub/scrub	B
71	Grassland/herbaceous	B
81	Pasture/hay	B
82	Cultivated crops	B
11	Open water	C
22	Developed, low intensity	C
23	Developed, medium intensity	C
24	Developed, high intensity	C
31	Barren land	C

Pairwise effective resistance between each sampling site was measured based on a circuit theory approach in GFlow (Leonard et al., 2017). We conducted partial Mantel tests with 10,000 permutations using the vegan R package to evaluate correlations between pairwise effective resistance values and genetic differentiation (*G*_ST_) while controlling for effects of geographic distance between sites. The candidate resistance cost values that explained the most variation (largest *R* ^2^ value) were selected as optimal. Four to seven resistance values were tested for each landscape feature. These values were selected based upon the results of Richardson (2012) and always included a value of 1 to allow comparisons between the candidate resistance values and a simple IBD relationship. All nonfeature raster cells were assigned a value of 1 during the optimization procedure, and the terrain ruggedness index was optimized by adding various resistance values to the actual index values.

Optimized cost surfaces were used to inform a series of generalized linear additive models to assess the relative contribution of each landscape feature to overall patterns of genetic differentiation among sites for each species. We only considered landscape variables that explained genetic diversity patterns better than IBD alone. Models were compared using the small sample size‐corrected Akaike's information criterion (AIC_C_; Burnham & Anderson, 2002). AIC_C_ compares relative support of candidate models including a penalty for the number of variables incorporated, thereby encouraging parsimony. Models with ΔAIC_C_ value <2 were considered equally supported. We used all possible combinations of included variables as candidate models and calculated AIC_C_ values and their relative weights using R package glmulti (Calcagno & Mazancourt, 2010).

## 3 RESULTS

### 3.1 Sampling and quality control

Due to a longer duration prior to hatching, all spotted salamanders were collected as embryos, reducing the likelihood of siblings being sampled, whereas wood frogs were occasionally collected as larvae. Therefore, sibship analyses and subsequent elimination of all but one member from each family group were performed only for the wood frogs. We sampled across multiple years for 31 spotted salamander and 27 wood frog populations. Allelic richness and expected heterozygosity did not differ depending on the number of years a site was sampled (*p* > .05). Resampled sites were never found to be unoccupied in any particular year. In total, we collected and genotyped 2,862 spotted salamander eggs and 2,935 wood frog eggs and larvae. Following removals of individuals based on genotype completeness, sample size, and sibship, 2,413 spotted salamanders from 90 sites and 2,439 wood frogs from 87 sites were included in our analyses (Table 3). Pairwise distances between sites ranged from 0.12 km to 320.55 km for spotted salamanders (mean = 120.32 km) and 0.07 to 337.88 km for wood frogs (mean = 120.47 km).

**Table 3:** Site location information, sample size, quality control, and genetic diversity information for 90 spotted salamander and 87 wood frog populations

Site name	Latitude	Longitude	Spotted salamanders							Wood frogs						
			*N*	HW	LD	*A*_O_	AR	*H*_E_	*F*_IS_	*N*	HW	LD	*A*_O_	AR	*H*_E_	*F*_IS_
ALF‐1	43.50788	−70.76709	17	0	0	6.63	5.73	0.723	0.045	23	0	0	11.30	5.25	0.855	0.067
AMH‐1	44.856017	−68.415817	15	0	0	6.75	6.05	0.755	−0.049	23	0	0	11.30	5.04	0.798	0.004
AMH‐2	44.831733	−68.41081	44	0	0	7.88	5.97	0.744	0.049	40	0	2	13.00	5.06	0.805	0.058
AMH‐3	44.8625	−68.396077	33	0	0	7.63	5.67	0.704	−0.053	17	0	0	10.40	5.18	0.831	−0.004
AMH‐4	44.855217	−68.411587	29	0	0	7.50	5.95	0.757	0.014	17	0	0	10.10	5.14	0.811	0.079
BAN‐1	44.863786	−68.736084	13	0	0	6.00	5.63	0.756	0.011	–	–	–	–	–	–	–
BAN‐2	44.79044	−68.83422	18	1	1	6.63	5.66	0.726	0.047	27	0	0	11.40	4.91	0.789	−0.033
BAN‐3	44.79794	−68.83723	–	–	–	–	–	–	–	19	0	2	10.50	5.11	0.816	0.193
BAN‐5	44.8632	−68.75721	30	0	0	7.50	5.78	0.740	0.070	–	–	–	–	–	–	–
BAN‐6	44.86372	−68.75248	30	0	0	7.00	5.55	0.723	−0.007	–	–	–	–	–	–	–
BAN‐7	44.80195	−68.78882	–	–	–	–	–	–	–	26	0	1	8.10	4.25	0.745	0.041
BEN‐1	44.58715	−69.51235	23	0	0	7.13	5.72	0.693	−0.046	43	0	3	13.60	5.29	0.837	−0.009
BRU‐1	43.89274	−69.98714	68	0	0	8.00	5.71	0.716	0.058	58	1	6	13.90	5.41	0.863	0.035
BRU‐2	43.935533	−70.008217	35	0	1	6.63	5.23	0.700	0.042	28	0	0	12.60	5.24	0.851	0.023
BRU‐3	43.916783	−69.98465	26	0	0	7.63	5.79	0.733	−0.009	74	1	1	15.40	5.42	0.861	0.048
BUX‐1	43.59865	−70.469383	23	0	0	7.38	5.85	0.742	0.048	33	0	2	13.40	5.27	0.847	0.009
CUM‐1	43.819133	−70.252717	32	0	1	7.38	5.75	0.733	0.062	62	2	1	14.00	5.46	0.876	0.056
DIX‐1	44.6857	−69.134633	28	0	0	7.63	5.85	0.714	−0.011	–	–	–	–	–	–	–
EDG‐1	43.97081	−69.58153	61	0	0	8.00	5.57	0.732	0.002	27	1	3	10.90	5.02	0.834	0.056
EDM‐1	44.8853	−67.2789	–	–	–	–	–	–	–	24	0	0	10.70	4.86	0.768	0.060
ELL‐1	44.60868	−68.36858	26	0	0	6.88	5.66	0.727	0.011	26	0	2	9.70	4.73	0.777	0.016
FAL‐1	43.80073	−69.75086	23	0	0	5.88	4.98	0.698	0.134	19	0	0	8.00	4.61	0.794	−0.028
FRA‐1	44.627483	−68.904683	30	0	0	7.25	5.60	0.672	0.032	23	0	1	10.80	5.02	0.823	0.054
FRE‐1	43.856667	−70.080133	13	0	0	5.88	5.41	0.685	0.078	15	0	0	9.80	5.43	0.854	0.051
FRE‐2	43.87105	−70.110917	43	0	0	7.50	5.73	0.744	0.031	61	1	1	14.90	5.27	0.853	0.055
FRE‐3	43.8005	−70.1331	22	0	0	6.88	5.51	0.693	0.040	18	0	0	9.60	4.92	0.831	0.081
GLS‐1	45.17524	−67.82938	25	0	0	6.75	5.50	0.690	0.009	27	0	0	10.80	4.89	0.774	0.014
GLS‐2	45.17594	−67.86995	29	0	0	6.75	5.33	0.688	0.088	–	–	–	–	–	–	–
GLS‐3	45.18357	−67.965	–	–	–	–	–	–	–	21	2	0	10.90	4.90	0.784	0.092
GRE‐1	45.440053	−69.392208	31	0	0	7.38	5.52	0.712	−0.006	–	–	–	–	–	–	–
GRE‐2	45.538783	−69.543654	23	0	0	7.25	5.93	0.722	−0.013	–	–	–	–	–	–	–
GRE‐3	45.695279	−69.46204	30	0	0	7.38	5.81	0.744	0.074	58	0	0	13.80	5.20	0.831	0.037
GRE‐4	45.708404	−69.669413	20	0	0	6.25	5.33	0.708	0.001	39	0	0	12.90	5.14	0.832	−0.003
HAM‐1	44.78044	−68.79021	39	0	0	7.50	5.88	0.747	0.058	50	0	4	12.00	5.02	0.797	−0.009
HAM‐2	44.87251	−68.70609	39	0	2	5.88	4.99	0.680	0.010	17	0	2	9.90	4.92	0.805	0.022
HAM‐3	44.725234	−68.839386	12	0	0	5.88	5.59	0.737	−0.028	19	0	0	11.00	5.18	0.826	0.013
HAM‐4	44.72599	−68.83836	23	1	0	6.50	5.60	0.743	−0.022	16	0	0	10.40	5.11	0.795	0.030
HAM‐5	44.72611	−68.83833	17	0	0	6.13	5.51	0.764	0.108	–	–	–	–	–	–	–
HAM‐6	44.76887	−68.81382	–	–	–	–	–	–	–	16	0	0	10.40	5.20	0.808	0.123
JEF‐1	44.13455	−69.5714	23	0	0	7.38	5.78	0.717	0.044	–	–	–	–	–	–	–
JEF‐2	44.13133	−69.5752	60	0	1	8.63	5.73	0.714	−0.043	31	0	0	12.80	5.28	0.841	0.035
JEF‐3	44.22778	−69.46122	64	0	0	8.88	5.98	0.714	−0.014	37	0	2	12.90	5.24	0.839	−0.003
JEF‐4	44.1574	−69.57211	23	0	0	7.13	5.89	0.740	0.060	30	0	1	12.70	5.30	0.837	0.034
JON‐1	44.70088	−67.5263	23	0	0	7.88	6.37	0.764	0.005	29	0	0	11.30	4.89	0.769	0.028
LIN‐1	44.33146	−68.063	17	0	0	6.25	5.62	0.702	0.146	–	–	–	–	–	–	–
LOV‐1	44.2119	−70.87366	29	0	0	7.63	5.85	0.705	0.018	23	1	0	13.00	5.41	0.865	0.042
LOV‐2	44.18099	−70.9356	15	0	0	6.13	5.64	0.719	−0.122	25	0	0	12.00	5.30	0.861	0.024
MAC‐1	44.72039	−67.5263	–	–	–	–	–	–	–	19	0	0	8.70	4.56	0.748	0.012
MNT‐1	44.44843	−69.30656	31	0	0	7.00	5.48	0.676	0.016	16	0	0	10.50	5.24	0.835	0.010
NEW‐1	43.9913	−69.5885	–	–	–	–	–	–	–	26	0	0	11.30	5.14	0.835	0.015
OGU‐1	43.24324	−70.61281	22	0	0	7.50	5.77	0.717	0.013	28	1	0	13.40	5.34	0.860	0.063
ORO‐1	44.888583	−68.78225	20	0	0	6.50	5.55	0.743	0.083	26	0	0	11.40	5.11	0.816	0.007
ORO‐3	44.878717	−68.757317	19	1	0	6.75	5.92	0.721	−0.024	–	–	–	–	–	–	–
ORO‐4	44.895533	−68.722867	29	0	3	7.50	5.79	0.757	0.064	32	0	1	10.70	4.91	0.801	−0.031
ORO‐5	44.8933	−68.723917	15	0	0	6.25	6.03	0.762	0.034	–	–	–	–	–	–	–
ORO‐6	44.889383	−68.760517	18	0	0	6.63	5.58	0.754	0.056	–	–	–	–	–	–	–
ORO‐7	44.8981	−68.68752	12	0	0	5.75	5.78	0.728	−0.088	12	0	0	8.80	4.99	0.791	−0.032
ORO‐8	44.87286	−68.70531	37	0	0	8.25	5.43	0.723	0.055	23	0	0	11.20	5.09	0.808	0.000
ORO‐10	44.89615	−68.72778	23	0	0	7.13	5.97	0.746	−0.014	–	–	–	–	–	–	–
OT‐2	44.93884	−68.68915	–	–	–	–	–	–	–	26	0	0	11.60	5.05	0.805	0.032
OT‐3	44.93901	−68.6713	19	0	0	6.88	5.82	0.706	−0.025	–	–	–	–	–	–	–
OT‐5	44.93901	−68.6713	–	–	–	–	–	–	–	21	0	0	11.20	5.15	0.807	0.015
PHI‐1	44.82591	−70.40891	20	0	0	6.75	5.86	0.718	0.064	22	0	0	12.10	5.39	0.853	−0.019
PIT‐1	44.79525	−69.3681	19	0	0	6.63	5.72	0.733	0.036	44	3	1	13.60	5.49	0.863	0.091
PRO‐1	44.536833	−68.880817	16	0	0	6.88	6.00	0.748	0.032	–	–	–	–	–	–	–
POW‐1	43.94315	−70.203017	–	–	–	–	–	–	–	28	0	0	12.50	5.32	0.856	−0.014
SBR‐1	43.87555	−69.56431	53	0	0	7.25	5.48	0.683	0.020	33	0	1	10.70	4.85	0.798	−0.008
SCA‐1	43.55003	−70.36169	32	1	2	6.50	4.97	0.702	0.051	30	0	4	10.30	4.95	0.832	0.031
SCA‐2	43.60163	−70.38015	24	0	0	6.13	5.16	0.699	0.026	31	1	0	12.70	5.34	0.851	0.060
SEA‐1	44.49271	−68.93039	–	–	–	–	–	–	–	26	0	2	10.10	4.93	0.805	−0.003
SEB‐1	43.90471	−70.67239	49	0	0	7.63	5.53	0.731	0.013	27	0	0	13.40	5.31	0.849	−0.017
SHA‐1	43.50199	−70.79466	20	0	0	7.00	5.86	0.745	0.139	29	0	0	12.00	5.18	0.834	0.049
SID‐1	44.42603	−69.70481	–	–	–	–	–	–	–	26	1	0	12.20	5.21	0.845	0.064
SKO‐1	44.7757	−69.74332	27	0	0	7.75	5.91	0.749	0.000	28	0	0	12.30	5.22	0.838	0.008
SKO‐2	44.76549	−69.59269	–	–	–	–	–	–	–	20	0	2	10.90	5.18	0.834	0.014
STA‐1	44.77255	−69.91174	16	0	0	6.25	5.56	0.716	0.000	38	0	0	13.60	5.31	0.842	0.050
STA‐2	44.77752	−69.92757	20	0	0	6.38	5.42	0.717	−0.015	19	0	0	10.80	5.30	0.858	0.058
SUL‐1	44.52482	−68.16853	22	0	2	6.88	5.67	0.721	0.001	22	0	1	10.20	4.96	0.792	0.033
TAT‐1	43.28084	−70.69041	51	0	0	7.63	5.71	0.732	0.028	18	0	0	11.00	5.13	0.830	−0.002
TOP‐1	43.954483	−69.976483	23	0	0	6.25	5.31	0.710	−0.027	30	0	0	11.60	5.00	0.821	0.018
TRE‐1	44.80536	−67.15285	–	–	–	–	–	–	–	12	0	0	7.50	4.41	0.736	0.021
WAL‐1	44.180917	−70.024217	15	0	0	7.00	6.14	0.752	0.008	45	0	0	13.80	5.36	0.852	0.043
WAY‐1	44.366033	−70.035417	33	0	0	7.25	5.58	0.702	0.055	58	0	4	14.50	5.21	0.838	0.011
WB‐1	43.74574	−70.36393	–	–	–	–	–	–	–	21	1	0	11.60	5.40	0.863	0.042
WEL‐1	43.34105	−70.55378	18	0	0	6.75	5.75	0.734	−0.013	40	0	0	13.40	5.22	0.845	0.018
WEL‐2	43.340982	−70.55058	31	0	3	7.13	5.63	0.747	−0.015	28	1	1	12.50	5.42	0.868	0.045
WEL‐4	43.31988	−70.59468	32	0	0	6.50	5.20	0.717	0.059	19	0	0	11.40	5.10	0.817	−0.030
WEY‐1	45.39568	−70.0009	–	–	–	–	–	–	–	15	0	0	10.40	5.28	0.842	0.047
WEY‐2	45.044343	−69.990037	11	0	0	5.25	5.11	0.657	−0.004	25	0	4	12.30	5.38	0.853	−0.080
WEY‐3	45.09518	−69.78388	28	0	0	7.50	5.48	0.728	−0.025	26	0	0	12.40	5.23	0.841	0.031
WG‐1	44.22255	−69.893033	11	0	0	6.00	5.84	0.722	−0.036	34	0	0	13.90	5.49	0.868	−0.023
WG‐2	44.221783	−69.901083	12	0	0	5.13	4.89	0.680	−0.026	–	–	–	–	–	–	–
WHI‐1	44.78317	−67.54623	19	0	0	7.38	6.04	0.745	0.061	30	0	1	11.40	4.91	0.768	−0.023
WIL‐1	44.61431	−70.26837	17	0	0	6.00	5.27	0.719	−0.107	23	0	6	10.20	5.21	0.855	0.033
WIS‐1	43.960033	−69.693833	–	–	–	–	–	–	–	29	0	1	11.40	5.22	0.851	0.015
WLM‐1	45.3036	−69.34457	24	0	0	6.25	5.41	0.724	−0.067	–	–	–	–	–	–	–
WM‐1	45.023405	−70.454206	–	–	–	–	–	–	–	13	0	4	9.00	5.29	0.847	0.143
WM‐2	44.92064	−70.50822	19	0	0	7.38	5.99	0.710	−0.097	12	0	0	9.50	5.28	0.851	0.056
WM‐3	44.767724	−70.49833	10	0	0	5.75	5.75	0.732	−0.158	–	–	–	–	–	–	–
WM‐4	44.73882	−70.45364	17	0	0	5.88	5.29	0.716	−0.023	30	0	0	13.60	5.45	0.867	0.012
WOL‐1	43.9494	−69.807	29	0	0	7.88	6.23	0.776	−0.087	28	0	0	12.10	5.17	0.828	0.033
WOO‐1	44.4036	−70.548683	21	0	0	7.00	5.71	0.733	0.022	29	0	0	13.20	5.45	0.869	0.014
YAR‐1	43.79115	−70.205483	14	0	0	6.75	6.00	0.706	−0.040	20	0	0	11.80	5.34	0.856	0.026
YAR‐2	43.821117	−70.209033	47	0	0	6.75	5.19	0.693	0.124	–	–	–	–	–	–	–
YAR‐3	43.98565	−69.944283	–	–	–	–	–	–	–	21	0	1	11.10	5.24	0.850	−0.033
YAR‐4	43.812283	−70.176717	–	–	–	–	–	–	–	16	0	4	9.20	5.02	0.835	−0.068
YOR‐1	43.122728	−70.64477	30	0	0	5.75	4.92	0.681	−0.044	–	–	–	–	–	–	–
YOR‐2	43.175521	−70.71255	32	0	0	7.13	5.29	0.692	0.002	24	0	0	12.30	5.34	0.852	0.013
YOR‐3	43.177481	−70.640711	25	0	0	6.75	5.71	0.759	0.015	–	–	–	–	–	–	–
YOR‐4	43.230644	−70.631586	57	1	0	7.88	5.43	0.711	0.022	–	–	–	–	–	–	–
Average			26.81	0.06	0.18	6.91	5.64	0.72	0.01	28.03	0.20	0.83	11.58	5.15	0.83	0.03

Abbreviations: *A*_O_, average number of alleles across loci; AR, allelic richness; *F*_IS_, Wright's inbreeding coefficient; *H*_E_, expected heterozygosity; HW, number of Hardy–Weinberg equilibrium nonconforming samples; LD, number of samples with loci in linkage disequilibrium; *N*, sample size.

Two spotted salamander loci had high null allele frequencies (AmaD328: 0.272 and AmaD315: 0.283) and were therefore excluded from further analyses. Null allele frequencies for the remaining eight spotted salamander loci ranged from 0.005 to 0.027. Tests of nonrandom assortment of genotypes indicated 16 of 2,578 tests (0.6%; Table 3) were significant. Significant violations of Hardy–Weinberg proportions were observed in 5 of 728 tests (0.7%; Table 3). Wood frog null allele frequencies ranged from 0.012 to 0.055 among loci. Tests of nonrandom assortment of genotypes indicated 72 of 3,897 tests (1.8%; Table 3) for wood frogs were statistically significant. Significant violations of Hardy–Weinberg proportions were observed in 17 of 870 tests (2.0%; Table 3) for wood frogs. No clear patterns of significance were detected within loci or sampling sites for either nonrandom assortment of genotypes or Hardy–Weinberg testing for either species; therefore, no loci or sites were excluded on the basis of these tests. *F*_IS_ averaged 0.01 (±0.0052 *SE*) for spotted salamanders and 0.03 (±0.0041 *SE*) for wood frogs (Table 3). Missing allele calls occurred for 1.3% of locus–sample combinations for spotted salamander and 2.1% for wood frogs. Genotyping error rates were observed in 0.8% of instances for wood frogs and 0.9% for spotted salamanders.

### 3.2 Genetic diversity, differentiation, and isolation by distance

**Figure 2:**
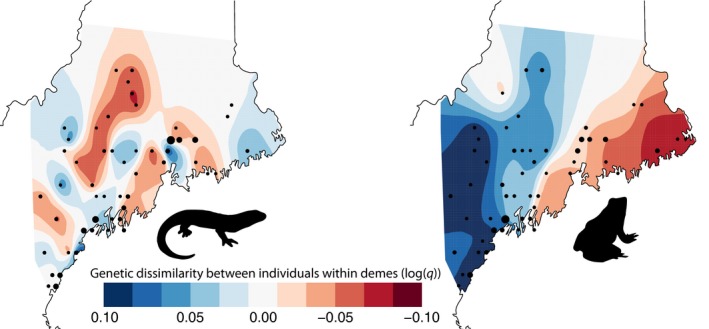
Spatially heterogeneous effective rates of genetic diversity among 54 spotted salamander and 53 wood frog demes. Black points indicate the location and relative sample size of each deme

Measures of genetic diversity, including *H*_E_, AR, and *F*_IS_, varied between the species but were of similar magnitudes. Across loci and among sites, spotted salamander AR averaged 5.64 (±0.298 *SE*) and *H*_E_ averaged 0.72 (±0.024 *SE*), whereas wood frog AR averaged 5.15 (±0.233 *SE*) and *H*_E_ averaged 0.83 (±0.032 *SE*; Table 3). The greater difference in values between species for AR relative to *H*_E_ is unsurprising given the relative insensitivity of *H*_E_ to the number of alleles observed (Maruyama & Fuerst, 1985). Following an FDR correction, no evidence of genetic bottlenecks was detected for either species at any population. EEMS analyses generated 54 spotted salamander and 53 wood frog demes. Strongly contrasting geographic patterns of genetic diversity were observed between the two species. For instance, across the range of study sites, spotted salamanders had several interspersed regions of high and low diversity, whereas wood frogs showed a clear gradient of high diversity to the west transitioning to lower diversity in the east (Figure 2). Because the analysis occasionally groups separate sampling sites into a single deme, and both species have strong spatial structuring, some locally high levels of diversity identified by the analysis may be due to the grouping of dissimilar populations into a single deme.

Average genetic differentiation among sites was relatively low for both species but varied widely. For spotted salamanders, global *G*_ST_ was 0.024 and *G″*_ST_ was 0.087. Pairwise *G*_ST_ values ranged from −0.006 to 0.068 and *G″*_ST_ ranged from −0.048 to 0.384. Following FDR correction of alpha levels, 3,474 of 4,005 tests (86.7%) were significant. For wood frogs, global *G*_ST_ was 0.032 and *G″*_ST_ was 0.189. Pairwise *G*_ST_ values ranged from −0.002 to 0.068, and *G″*_ST_ ranged from −0.029 to 0.583. Following FDR correction of alpha levels, 3,639 of 3,741 tests (97.3%) were significant. All pairwise *G*′_ST_ and *G″*_ST_ values are provided in Appendix S2.

**Figure 3:**
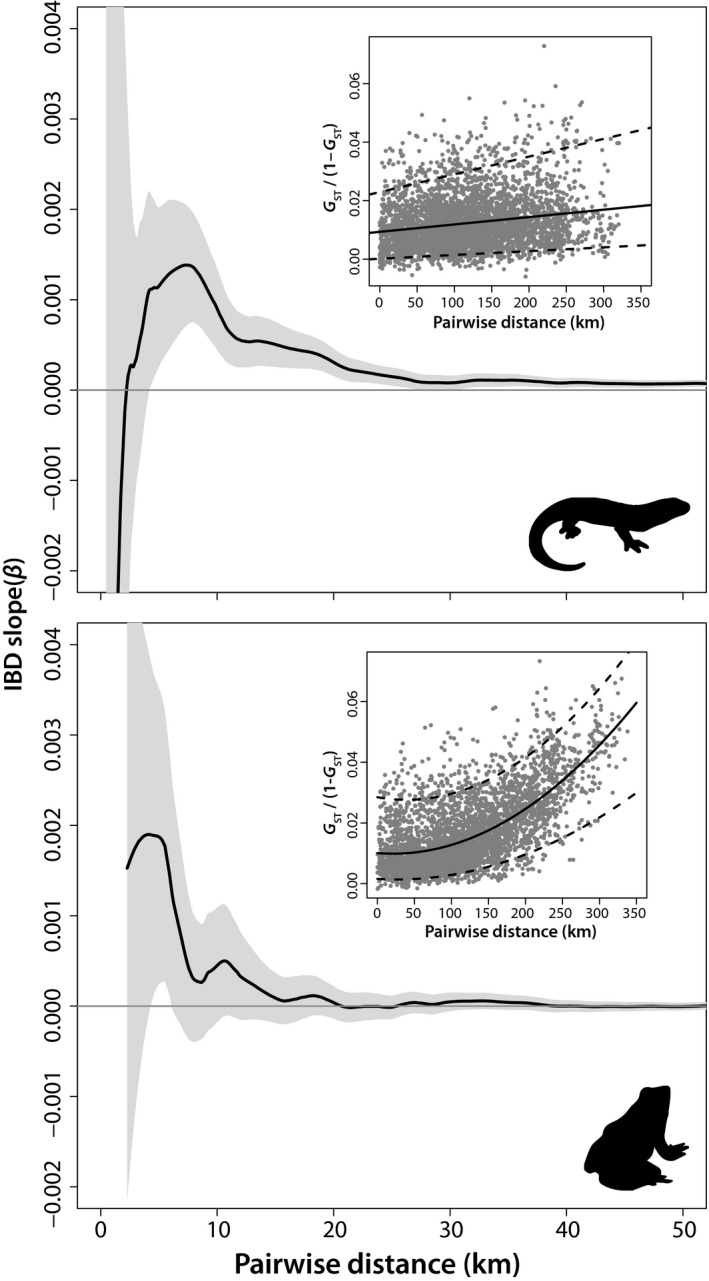
Associations between the slope (*β*) of the regressed isolation‐by‐distance (IBD) relationship and the maximum pairwise distance of the sample set considered for spotted salamanders and wood frogs. Shaded areas indicate the 95% confidence intervals of β coefficients for each iteration of the analysis. Inset figures depict pairwise relationships between geographic (km) and linearized genetic distances (*G*_ST_/1−*G*_ST_) indicated by ordinary least squares (OLS) regression (solid line) and 95th and 5th quantile regressions (dashed lines) for 90 spotted salamander and 87 wood frog populations. Wood frog OLS regression: *y* = 1.028 × 10^−2^ + −1.831 × 10^−5^ *x* + 4.581 × 10^−7^ *x* ^2^, *R* ^2^ = 0.445, Mantel's *r* = 0.628, *p* < .001. Spotted salamander: *y* = 9.416 × 10^−3^ + 2.535 × 10^−5^ *x*, *R* ^2^ = 0.038, Mantel's *r* = .196, *p* < .001

Isolation‐by‐distance patterns differed between the two species. Despite relatively weak correlation using each genetic distance, IBD relationships were statistically significant for spotted salamanders based on nontransformed geographic distances (*G*_ST_: *r* = .196, *p* < .001; and *G″*_ST_: *r* = .203, *p* < .001), as well as following log transformation (*G*_ST_: *r* = .18, *p* < .001; and *G″*_ST_ *r* = .181, *p* < .001). IBD patterns without the geographic distance log transformation were stronger for wood frogs for *G*_ST_ (*r* = .628, *p* < .001) and *G″*_ST_ (*r* = .593, *p* < .001) and were marginally weakened following transformation for both *G*_ST_ (*r* = .461, *p* < .001) and *G″*_ST_ (*r* = .433, *p* < .001). Because the IBD relationship for the wood frogs appeared nonlinear, we also fit a quadratic rather than linear model to the data. Due to similar patterns between the genetic distance measures, only plots based on *G*_ST_ are shown (Figure 3 inset panels). The regression slopes for genetic distance versus log‐transformed geographic distances were significantly different from zero for both species (*p* < .001), and the slope estimate for spotted salamanders (*β* = 0.0018) was less than that for wood frogs (0.0064).

**Figure 4:**
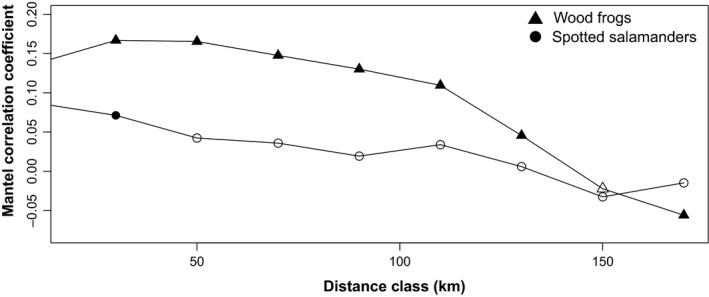
Mantel correlograms indicating associations between genetic (*G*_ST_) and geographic distance among spotted salamander and wood frog site pairs. Filled symbols indicate statistical significance based on 10,000 bootstrap replicates and a false discovery rate correction for multiple testing based on an alpha level of 0.05

**Figure 5:**
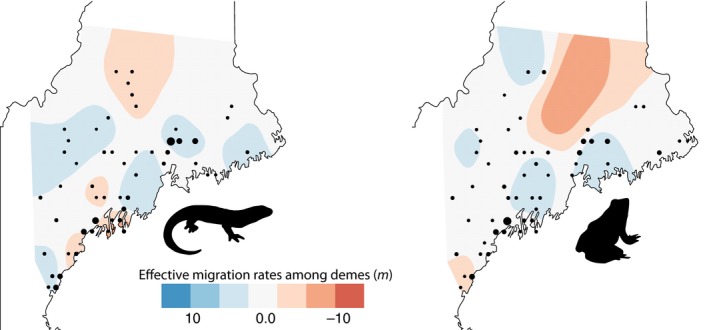
Spatially heterogeneous effective rates of migration among 54 spotted salamander and 53 wood frog demes. Black points indicate the location and relative sample size of each deme. Migration rates (*m*) are relative to background rates. For instance, a value of 10 is reflective of 10× greater migration than the background rate

Our IBD scaling profiles indicated that *β* values ranged widely for each species depending on the maximum pairwise distance included in the analysis and the responses of β to maximum pairwise distances were strongly nonlinear for both species (Figure 3). The Mantel correlogram indicated that IBD relationships were strongest at shorter distance classes for both species, with spotted salamander associations becoming nonsignificant at distances greater than 60 km (Figure 4). For wood frogs, the greatest distance class had significant negative spatial autocorrelation, which aligns well with the particularly high levels of population differentiation at large scales that were detected with IBD regressions. EEMS identified several geographic regions with more and less gene flow than expected under an IBD scenario. For instance, the north‐central portion of the study area consistently had relatively low connectivity, whereas multiple coastal regions were more connected. An area of low connectivity was also noted for spotted salamanders in the most densely human‐populated area around Portland, Maine; however, a similar pattern was not observed for wood frogs (Figure 5).

### 3.3 Regression and multivariate analyses

We detected significant relationships among genetic diversity, urbanization, and isolation. Residuals were measured using the relationship among linearized *G*_ST_ and nontransformed geographic distance because a strong correlation with residuals of the log‐transformed relationship was observed for both species (spotted salamanders: 0.994; wood frogs: 0.934). Sampled sited represented a broad range of the measured environmental attributes (Appendix S3). Nearby canopy cover, distance to roadway, and nearby amount of impervious surface were all highly correlated with the total distance of light roads within 1 km of each study site, allowing us to retain only the three road classes. Multiple regression models that sought to explain variation in allelic richness and expected heterozygosity based on the three road classes were generally nonsignificant. Statistical significance was detected for the wood frog allelic richness model, and the secondary roads term had a significant positive relationship with expected heterozygosity for spotted salamanders, although each of these relationships explained very little variation, suggesting limited biological relevance (Table 4).

**Table 4:** Results of multiple regression models assessing effects of three road types on allelic richness, expected heterozygosity, and site isolation

Parameter	Allelic richness				Expected heterozygosity				Site isolation			
	Spotted salamanders		Wood frogs		Spotted salamanders		Wood frogs		Spotted salamanders		Wood frogs	
	*β*	*p*	*β*	*p*	*β*	*p*	*β*	*p*	*β*	*p*	*β*	*p*
Light roads	−1.78 × 10^−5^	.099	−9.82 × 10^−6^	.103	−1.20 × 10^−6^	.173	1.33 × 10^−7^	.871	**5.67 × 10^−7^**	**.003**	**5.99 × 10^−^^7^**	**<.001**
Secondary roads	8.95 × 10^−6^	.768	−1.91 × 10^−5^	.399	**5.22 × 10^−6^**	**.037**	−5.24 × 10^−6^	.093	1.44 × 10^−7^	.781	1.75 × 10^−8^	.968
Primary roads	−2.97 × 10^−5^	.202	−1.79 × 10^−5^	.319	−2.30 × 10^−7^	.903	−2.86 × 10^−6^	.245	−5.31 × 10^−8^	.893	2.75 × 10^−7^	.431
Adjusted *R* ^2^	0.026		0.061		0.024		0.026		0.086		0.297	
Full model *p*	.155		**.041**		.170		.162		**.013**		**<.001**	

Note

Variables and models with statistically significant values (*p* < .05) are indicated in bold font.

**Figure 6:**
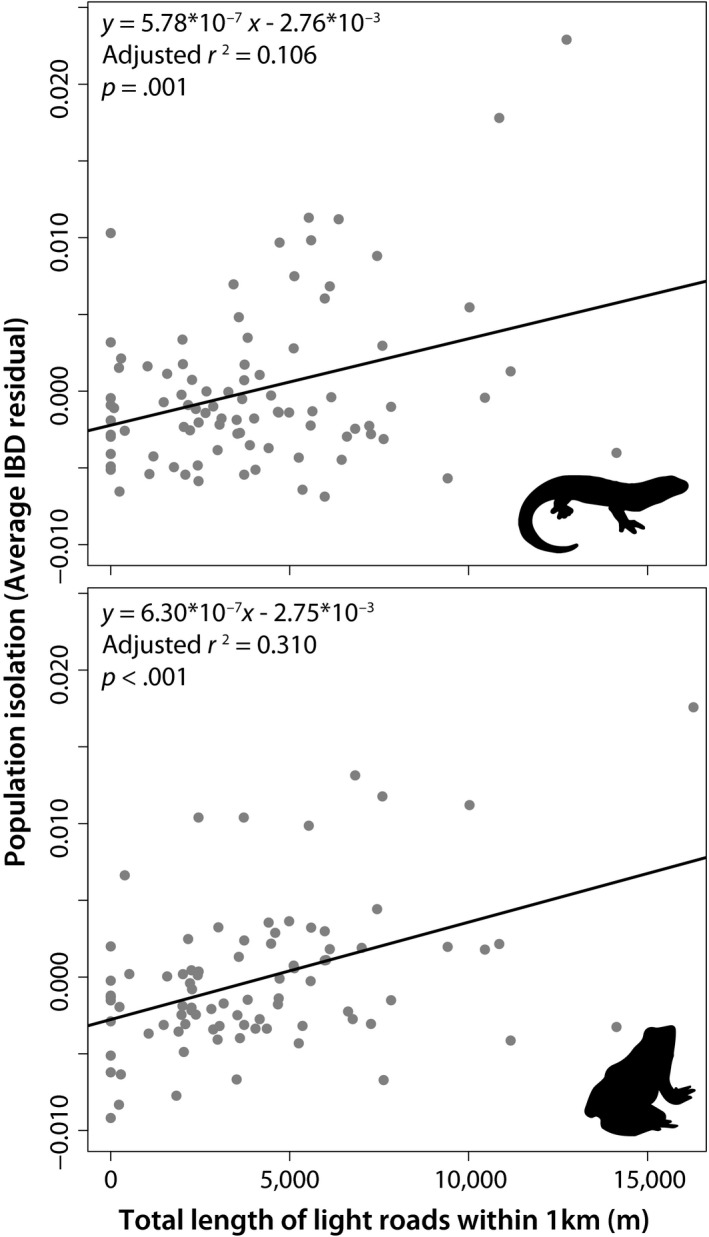
Linear regression analyses indicating a positive relationship between site isolation and the length of light roads within one km of a breeding site for spotted salamanders and wood frogs

**Figure 7:**
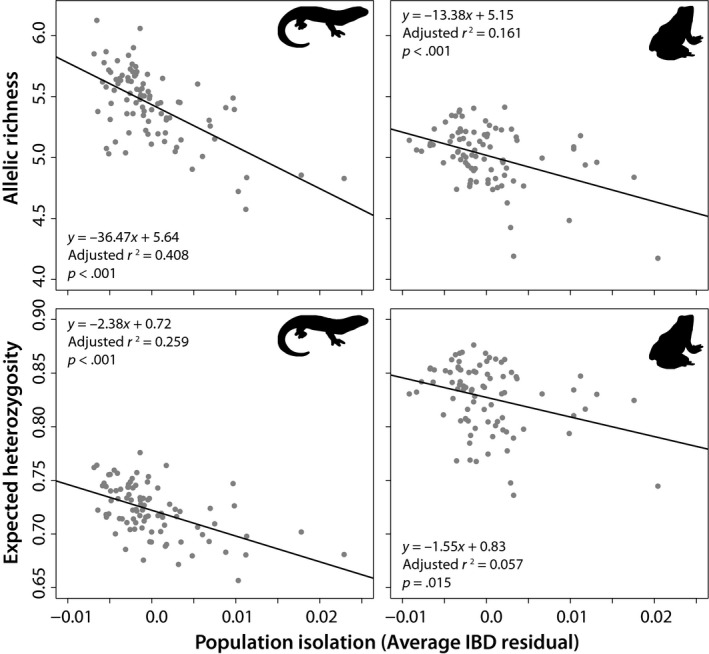
Linear regression analyses illustrating negative relationships between sampling site isolation and allelic richness and expected heterozygosity for analyzed spotted salamanders and wood frogs

The degree of isolation (mean IBD residual) experienced by a site was significantly greater for locations with more nearby light roads for both species, with this effect being stronger for wood frogs (*β* = 5.99 × 10^–7^, *p* < .001) than spotted salamanders (*β* = 5.67 × 10^–7^, *p* = .003; Table 4). For the wood frog dataset involving nearby light road length, a single outlier site was removed due to having over twice the distance of nearby light roads than the next closest site. The influence of light roads on site isolation was also analyzed using a simple linear regression (Figure 6), which further enforced the positive relationship. Finally, expected heterozygosity and allelic richness declined as a site's degree of isolation increased for both species; however, these declines were stronger for spotted salamanders than for wood frogs (Figure 7).

Distance‐based RDAs for each species identified relationships between interpopulation genetic differentiation and measures of urbanization. Following backward optimization, each model contained the light roads variable while controlling for latitude and longitude. Density of light roads was significantly associated with *G*_ST_ values for wood frogs (*F* = 3.95, *p* = .007), but statistical support for the spotted salamander model was marginal (*F* = 1.59, *p* = .097). Variance partitioning based on the RDAs' adjusted *R* ^2^ values revealed a much better overall model fit for wood frogs than spotted salamanders, mostly attributable to a stronger IBD signal for the wood frogs. For spotted salamanders, the light roads explained 2.4%, geography explained 5.4%, and the terms collectively explained 12.7% of the variation. For wood frogs, the amount of variation explained was 3.6% for light roads, 47.6% for geography, and 53.7% for the combined terms. MMRR further supported the existence of a significant relationship between interpopulation genetic divergence and both geographic distance and density of nearby light roads for both species (Table 5).

**Table 5:** Results of multiple matrix regression with randomization assessing effects of three road types on interpopulation genetic differentiation values

Parameter	Spotted salamanders			Wood frogs		
	*β*	*t*	*p*	*β*	*t*	*p*
Light roads	**6.12 × 10^−7^**	**11.93**	**.006**	**5.31 × 10^−7^**	**18.39**	**<.001**
Secondary roads	4.53 × 10^−^^8^	0.36	.921	2.22 × 10^–7^	1.69	.549
Primary roads	2.08 × 10^−^^7^	2.66	.616	5.76 × 10^–7^	7.24	.123
Geographic distance	**2.06 × 10^−^^5^**	**10.22**	**<.001**	**1.07 × 10^–4^**	**52.64**	**<.001**

Note

Variables with statistically significant values (*p* < .05) are indicated in bold font.

### 3.4 Landscape resistance

The resistance surface models that we constructed provided insight into the relative influence of numerous natural and anthropogenic landscape features on interpopulation connectivity for spotted salamanders and wood frogs. Optimization of resistance surfaces indicated several differences in which landscape features most influence connectivity for each species (Table 6; Appendix S4). For instance, light roads and interstates were suggested as important for spotted salamanders but not for wood frogs. Both river classes had less influence on genetic structure than distance alone for spotted salamanders, whereas rivers were strongly influential for wood frogs. Generally, anthropogenic features such as roads (particularly interstate highways) and developed land cover trended toward negative influences on connectivity in both species.

**Table 6:** Landscape features included in resistance surface models and optimized resistance values based on partial Mantel testing

Landscape feature	Spotted salamanders	Wood frogs
Land cover A	1	1
Land cover B	1	1
Land cover C	5	15
Interstates	1,000	500
Secondary roads	1	25
Light roads	25	10
Medium rivers	1	500
Large rivers	1	4,500
Railroads	1	1
Terrain ruggedness index	TRI + 500	TRI + 500

Note

Values of 1 represent an isolation‐by‐distance model.

Generalized linear modeling based on effective resistance distances between populations provided strong support for the influence of multiple landscape features on connectivity of each species. By limiting inclusions of landscape features to only those with a stronger influence than distance alone, we assessed four variables for spotted salamanders and seven variables for wood frogs, in addition to an IBD‐only model. This resulted in 17 candidate models for spotted salamanders and 129 for wood frogs. Spotted salamander resistance values were best explained with a single top model that included land cover class C (development and open water), interstates, light roads, and terrain ruggedness (Table 7). For wood frogs, there were four models within two AIC_C_ points of each other, indicating they were equally strongly supported. These four models each included land cover class C, medium rivers, large rivers, light roads, and terrain ruggedness with interstates and secondary roads each occurring in two of these top four models (Table 7). The top eight models all included land cover class C, and terrain ruggedness was present in the each of the top 12 models, suggesting a major role for these features in determining genetic structure for wood frogs. The IBD‐only model was one of the least supported for both species.

**Table 7:** Results of additive landscape resistance models ranked based on the parsimony‐weighted AIC_C_

Landscape resistance model	AICC	ΔAICC	Weight
Spotted salamanders			
LcC + Interstates + LtRoads + TRI	−26,456.06	0.00	0.94
Interstates + LtRoads + TRI	−26,450.33	5.73	0.05
Interstates + LtRoads	−26,443.78	12.28	0.00
LcC + Interstates + LtRoads	−26,443.74	12.32	0.00
LcC + LtRoads	−26,383.67	72.39	0.00
LtRoads + TRI	−26,381.98	74.07	0.00
LcC + LtRoads + TRI	−26,381.77	74.29	0.00
LtRoads	−26,378.66	77.40	0.00
LcC + Interstates + TRI	−26,254.38	201.67	0.00
LcC + Interstates	−26,215.35	240.71	0.00
Interstates	−26,206.53	249.53	0.00
Interstates + TRI	−26,204.55	251.51	0.00
Wood frogs			
LcC + LtRoads + MedRivers + LgRivers + TRI	−23,885.32	0	0.32
LcC + Interstates + LtRoads + MedRivers + LgRivers + TRI	−23,884.73	0.59	0.24
LcC + SecRoads + LtRoads + MedRivers + LgRivers + TRI	−23,884.19	1.13	0.18
LcC + Interstates + SecRoads + LtRoads + MedRivers + LgRivers + TRI	−23,883.61	1.71	0.14
LcC + MedRivers + LgRivers + TRI	−23,881.90	3.41	0.06
LcC + SecRoads + MedRivers + LgRivers + TRI	−23,880.89	4.43	0.03
LcC + Interstates + MedRivers + LgRivers + TRI	−23,879.95	5.37	0.02
LcC + Interstates + SecRoads + MedRivers + LgRivers + TRI	−23,878.94	6.37	0.01
SecRoads + LtRoads + MedRivers + LgRivers + TRI	−23,784.06	101.25	0.00
Interstates + SecRoads + LtRoads + MedRivers + LgRivers + TRI	−23,782.06	103.26	0.00
LtRoads + MedRivers + LgRivers + TRI	−23,780.59	104.72	0.00
Interstates + LtRoads + MedRivers + LgRivers + TRI	−23,778.59	106.73	0.00

Note

The top 12 ranked models are shown. Models only included variables with resistance values that explained genetic distances better than the null isolation‐by‐distance scenario.

Abbreviations: Interstates, interstate highways; LcC, land cover class C; LgRivers, sixth‐ and seventh‐order rivers; LtRoads, light roads; MedRivers, fourth‐ and fifth‐order rivers; SecRoads, secondary roads; TRI, terrain ruggedness index.

## 4 DISCUSSION

Natural and anthropogenic landscape features contribute to interpopulation genetic structuring for both spotted salamanders and wood frogs; however, the strength of effects for specific landscape features differs markedly between the species. Both species experience increased population isolation in urban areas and decreased genetic diversity as population isolation increases; however, no direct connection between roads and genetic diversity is evident. Moreover, specific natural and anthropogenic landscape features generally affect each species' gene flow differently, which is likely a result of how differing life history and behavioral tendencies influence interactions of each species with the landscape. These local effects also occur in the context of contrasting broadscale patterns of the distribution of genetic variation for each species. Collectively, these results suggest that urban landscape elements are reshaping metapopulation‐level dynamics for spotted salamanders and wood frogs, although the effects are not necessarily consistent among these two sympatric species.

### 4.1 Effects of urbanization

Our work has identified elements of urban landscapes that are capable of influencing connectivity among spotted salamander and wood frog populations. Density of light roadways was identified in multiple analyses as an important factor in restricting connectivity among populations. However, it is important to put this result in the context of a high level of correlation among light roads, canopy cover, distance to nearest road, and amount of nearby impervious surface. As such, the effects of light roadways are likely an indicator of urbanization as a whole, rather than light roads exclusively. However, the effects of roadways themselves on gene flow should not be understated, as they have consistently been recognized as hazardous for migratory amphibian species (reviewed in Schmidt & Zumbach, 2008) and previously observed to diminish interpopulation connectivity for spotted salamanders (Coster, Babbitt, Cooper, et al., 2015; Richardson, 2012) and wood frog (Richardson, 2012).

Estimated effective migration surfaces and resistance surface modeling revealed distinctive effects of several landscape features on gene flow for spotted salamanders and wood frogs. For instance, our resistance surface analyses indicated very strong effects of rivers on wood frog connectivity, but no detectable effect for spotted salamanders. Interstate highways, another modeled linear landscape feature, were found to have very strong effects on both spotted salamander and wood frog connectivity, which was unexpected given the interstates in Maine have only been in place for 60 years or less (Ferris, 1979). Relatively rapid responses in genetic structure to the presence of roadways have been observed in other species, but the effect is inconsistent (Holderegger and Di Giulio, 2010). Wood frog gene flow generally conformed to our hypothesis of stronger effects of natural (rivers, terrain ruggedness) versus anthropogenic features (roadways, developed landscapes), whereas salamander gene flow did not, with terrain ruggedness being the only natural landscape feature with an influence appreciably greater than distance. EEMS gene flow models (Figure 5) indicated several clear differences between species. For instance, spotted salamanders had relatively little gene flow where several peninsulas were sampled in the south‐central region of the coast, an area that also coincides with some of the most dense urban development (Figure 2). Wood frogs did not share this pattern, instead having a large area of restricted gene flow in the north‐central region of the study area.

Comparing our study with previous work highlights the context dependency of landscape genetic inferences. For instance, our results often contrast those of Richardson (2012), who used similar landscape genetics approaches with the same two species in the Connecticut River Valley, a region approximately 250 km to the southwest of our study range. That study found a lower IBD slope for wood frogs relative to spotted salamanders; however, the maximum geographic extent of our study was greater (approximately 350 km vs. 225 km), and we found the strongest differentiation for wood frogs occurred at geographic distances greater than those examined by Richardson (2012). Because Richardson (2012) found wood frog slopes were less than slopes for spotted salamanders, he suggests that gene flow likely occurs more frequently for wood frogs than spotted salamanders, whereas our results suggest that such a conclusion likely reflects spatial context. Our resistance surface modeling results also often contrasted with those presented by Richardson (2012), who identified a strong influence of medium and large rivers on spotted salamander structuring and strong effects of railways on both species, whereas we found no detectable effect. Regional differences in the correlation of various landscape features could lead to different variables having stronger or weaker relationships to resistance, and it is likely that real‐world resistance results from compounding factors that are not easily isolated. For instance, Richardson (2012) occurred in a topographically diverse environment, where railways likely follow specific elevation contours, creating collinearity between slope and railway variables that would not be present in most of our study region. Similar disparities among the influence of specific landscape features have been observed across the range of other species. For instance, Cope's giant salamander (*Dicamptodon copei*) had varying responses to waterways and forest cover in different areas of its range (Trumbo, Spear, Baumsteiger, & Storfer, 2013). Short Bull et al. (2011) found variable effects of the same landscape features across 12 black bear study areas in Montana and Idaho. That study also found that the most variable features within a study area were more likely to receive support in their model. Future studies or efforts to manage landscapes for resistance that focus on features identified in other regions may overlook locally important features and contribute to incomplete or ineffective management. This shortcoming may be addressed through replicated study designs, whereby separate areas of a species' range are the units of replication, or through separate studies as demonstrated here via our comparisons with Richardson (2012). A standardized procedure for quantifying landscape heterogeneity would also improve researchers' ability to make equitable comparisons among species and landscapes.

Inferences from resistance surface modeling are sensitive to the spatial scale (i.e., grain size and study extent) of analyses. Previous work has demonstrated a relationship between the grain size and estimated resistance values of environmental variables (Zeller et al., 2014); however, very high sensitivity was uncommon and even when observed, it remains difficult to determine which grain sizes are most ecologically meaningful. Our grain size was 90 m, which is reasonable given the home range of these species. Moreover, the grain size was determined by the resolution of our most coarse raster dataset and represented a trade‐off with the large study extent to maintain computational tractability. The effects of study extent on resistance estimates are less well resolved (Zeller, McGarigal, & Whiteley, 2012). Given that our study extent was many orders of magnitude greater than the study species' range size, we likely captured some effects unrelated to short‐term dispersal. In that case, the effects of discrete landscape elements (e.g., roadways or rivers) are likely underestimated, as their effect would be diluted across the greatest analyzed geographic distances. The inclusion of multiple species in our analyses should buffer any spurious outcomes associated with our chosen spatial grain and extent (Richardson et al., 2016).

We used the IBD residuals to inform an index for population isolation, which provides a pairwise genetic distance measure standardized using geographic distance and a means of determining the factors that contribute to population‐wise departures from an IBD pattern. Using this metric, we identified a significant relationship between isolation and the urbanization indicator of nearby density of light roads for both species, which was further supported by the elevated resistance values assigned to light roads (Table 6) and the significant influence of light roads identified in our dbRDA. We also used this isolation metric to detect relationships between increasing levels of isolation and declining levels of genetic diversity for both species. A similar relationship was observed by Crawford et al. (2016) who quantified the connectivity among populations of the pool‐breeding Jefferson salamander (*Ambystoma jeffersonianum*) and found that less connectivity among populations resulted in significantly lower heterozygosities and allelic richness. Similarly, Cosentino, Phillips, Schooley, Lowe, and Douglas (2012) found populations of tiger salamander (*A. tigrinum*) that were smaller and more isolated had less genetic diversity than populations that were more connected to one another. This lack of a direct effect between genetic diversity and urbanization is not theoretically surprising considering some gene flow likely still occurs, and a lag is likely present between the time for urbanization to generate isolation and the subsequent effects of that isolation on loss of diversity through drift. Along those lines, it is reasonable to hypothesize that much of the reduced genetic variation with isolation in our dataset comes from natural isolation patterns on the landscape. However, genetic diversity losses associated with urbanization have been observed in other species (e.g., Munshi‐South, Zolnik, & Harris, 2016), although the effect is not ubiquitous. For instance, dwarf salamanders (*Eurycea quadridigitata*) experienced reduced allelic richness as nearby road density increased, although the effect was absent for southern leopard frogs (*Lithobates sphenocephalus*) in the same landscape (McKee, Maerz, Smith, & Glenn, 2017).

### 4.2 Broadscale genetic structure

The spotted salamander IBD relationship has high variance and a low y‐intercept (Figure 3 inset), making it unusual among commonly observed patterns (Phillipsen et al., 2015; Hutchison & Templeton, 1999). Similar patterns have been observed for spotted salamanders throughout their range, including in central Massachusetts (Whiteley et al., 2014), central Missouri (Burkhart et al., 2017; Peterman et al., 2015), and northeastern Ohio (Purrenhage et al., 2009). Other studies that did not report an IBD intercept did find high variance relationships (Coster, Babbitt, & Kovach, 2015; Zamudio & Wieczorek, 2007). One potential explanation for high variance IBD patterns is an increase in the influence of genetic drift due to consistently depressed effective population sizes caused by limited recolonization capacity associated with relatively small dispersal distances for spotted salamanders. However, the small intercept value suggests an appreciable role of gene flow, and our effective population size estimates had confidence intervals consistently including infinity, likely due to an insufficient number of individuals or loci being sampled per population (analyses not shown). An unidentified factor in the species' biology or ecology such as exceptionally high microsatellite mutation rates or undocumented dispersal processes may also be contributing to the observed pattern.

The strongly nonlinear IBD pattern observed for wood frogs was unexpected and is not typically observed. Generally, nonlinear IBD relationships have been suggested to indicate departures from dispersal–drift equilibrium, secondary contact, or a colonization event (Bradbury & Bentzen, 2007; Hutchison & Templeton, 1999). However, most observed and simulated nonlinear IBD relationships follow a pattern of decreasing slope as geographic scale increases, rather than the increasing slope that we observed (Bradbury & Bentzen, 2007). The observed IBD pattern may result from a combination of contemporary and past processes. First, the absolute range boundaries along one edge of our sampling area created by the Atlantic Ocean may be inflating the degree of genetic differentiation experienced for populations near the boundary. Those populations can be expected to have fewer potential sources for inbound dispersers, possibly limiting the overall contribution of gene flow to homogenizing allele frequencies among populations and amplifying the effects of genetic drift or selection (Eckert, Samis, & Lougheed, 2008). In our case, such boundary effects may be particularly strong with several of the most distant wood frog sampling sites located along the coastline (Figure 1), meaning both populations in the most spatially distant paired comparison may be experiencing this effect. However, if genetic drift alone were driving differentiation, we should see an increase in *G*_ST_ variance for these sites as some drift to allele frequencies more similar to geographically distant sites (i.e., Hutchison and Templeton's (1999) case IV pattern). The observed pattern may also be influenced by postglaciation or postdeforestation recolonization patterns. For instance, recolonization of the region from multiple refugia may cause our most distant contemporary sampling sites to exhibit relatively high levels of differentiation, while secondary contact has eroded these differences for more centrally located sites (Durand, Jay, Gaggiotti, & François, 2009). Another consideration is that an increasing IBD slope may be more common than currently recognized and was only revealed here by the large number of sampled populations and the broad sampled extent relative to the species' dispersal distance (Table 1; Jenkins et al., 2010; Koen, Bowman, Garroway, & Wilson, 2013). Without concentrating sampling on the periphery of a study region, IBD data are inherently scant near study margins due to fewer possible pairwise population combinations at the largest distance classes, which causes anomalously high variance at the upper end of the distance distribution and potentially obscures ecologically relevant patterns.

We identified strong relationships between the spatial extent of analyses and the strength of IBD relationships using our IBD scaling profiles. By quantifying the IBD slope across the range of distance values in our dataset, we could assess the relative importance of gene flow to genetic structuring across scales for each species. Slopes were greatest for wood frogs up to about 6 km and to about 9 km for spotted salamanders, suggesting that opportunities for instances of substantive pairwise isolation and divergence quickly increase with distance as the strongest locally constraining effects of gene flow become less universal to all population pairs. Beyond these distances of inclusion, IBD slopes decrease as the incidence of isolated pairs that are substantively divergent becomes increasingly marginal. An analogous pattern of a strong influence of gene flow at relatively short distances was identified by van Strien, Holderegger, and Heck (2015), who performed a similar analysis to identify the distance of maximum correlation using simulated data. This result also emphasizes the importance of considering spatial scaling of inferences in landscape genetic studies, an issue that has recently been emphasized by other authors (Balkenhol et al., 2016). Cushman and Landguth (2010) conducted a series of Mantel tests to examine relationships between genetic and geographic distances using simulated data while varying the window size (i.e., extent) of their analysis. In their study, Mantel *r* values declined as window size increased, although the transition was relatively gradual. In our study, the slope of the regressed IBD relationship experienced significant nonlinear dynamics depending on the spatial extent that was evaluated. If such scaling patterns are prevalent for IBD, which seems likely, this would suggest that there is little validity in directly comparing overall IBD slopes across studies conducted at very different geographic scales. Although more in‐depth analyses are possible (e.g., Galpern & Manseau, 2013), a workable alternative is to use the provided R code to generate IBD scaling profiles (see Appendices [Link], [Link], [Link], [Link]) for comparison of different studies or species at overlapping IBD inference scales.

## 5 CONCLUSIONS

Our study identified critical differences and similarities in how two sympatric species with similar habitat requirements are affected by landscape context. At the scale of single populations, both species responded negatively to the effects of nearby urbanization, whereas interpopulation dynamics differed between the species depending on landscape features. These results can inform conservation of pool‐breeding amphibians, as well as metapopulation‐structured species more broadly. Species with small home ranges capable of satisfying all their life history requirements are sometimes protected using a core–habitat conservation approach (Baldwin, Calhoun, & de Maynadier, 2006a; Semlitsch & Jensen, 2001) that aims to protect species through preserving the structures and functions of requisite habitats. This approach is often applied to pool‐breeding amphibians, where breeding habitats and adjacent upland environments are targeted for protections (Baldwin et al., 2006b). While this conservation strategy protects the majority of individuals that are faithfully philopatric to natal sites (Vasconcelos & Calhoun, 2004), the dispersers that demographically and evolutionarily connect subpopulations are left vulnerable if the intervening landscape is unprotected. The importance of landscape protections aimed at preserving connectivity among subpopulations has been recognized and implemented for some large‐bodied species (e.g., establishment of wildlife corridors; Sharma et al., 2013), and a landscape genetic approach as applied here is well positioned to provide insight into dispersal routes for more cryptic species. Preservation or restoration of those landscape types that are highly permeable to gene flow could be particular effective for increasing metapopulation‐level stability in amphibian species, as a loss of connectivity among populations has been identified as a leading cause of biodiversity loss (Pittman, Osbourn, & Semlitsch, 2014), which is occurring worldwide (Dudaniec, Spear, Richardson, & Storfer, 2012; McCallum, 2007). Assessing the habitat and corridor requirements of sympatric species could also improve the efficacy of management actions by identifying features that similarly affect multiple species. In our case, similarly strong effects of interstate highways indicate that mitigation efforts targeted toward large roadways (e.g., wildlife underpasses or overpasses; Hamer, Langton, & Lesbarreres, 2015) may provide the strongest return on investment for management actions. However, given contrasting results between our study and those of Richardson (2012), the generalizability of these inferences in the context of regionally dependent correlates that may drive observed relationships is warranted.

This research also underscores the importance of scale dependency when considering spatially explicit interpopulation relationships, as highlighted by our IBD scaling profiles that revealed strong spatial variation in isolation‐by‐distance relationships. Research geared toward quantifying variation in gene flow across other studies (e.g., using IBD *β* values) would benefit from considering the extent over which the data were collected to generate equitable comparisons. Quantifying such variation can help provide an understanding of the heterogeneity in a species' dispersal propensity, consequently producing a more realistic understanding of how species interact with their surrounding environments. Overall, this research provides a strong example of the capacity of urbanization to shape species' interpopulation dynamics. A deeper understanding of the causes and consequences of these effects will provide a robust foundation for identifying and mitigating current and future risks to biodiversity.

## CONFLICT OF INTERESTS

None declared.

## AUTHOR CONTRIBUTIONS

JJH, CSL, and MTK designed the study. JJH collected samples, performed laboratory work, and conducted data analyses. JJH led the writing of the manuscript. All authors contributed critically to the drafts and approved of the final manuscript.

## Data Availability

All R code and data associated with this manuscript are available individually and as an R package at https://github.com/jaredhomola/VPLandscapeGenetics, which has been archived on Zenodo at https://zenodo.org/record/3403056. Genotypic, environmental, and site location data are also available on Dryad https://doi.org/10.5061/dryad.d59cq77.

## References

[ece35685-bib-0001] Adamack, A. T. , & Gruber, B. (2014). PopGenReport: Simplifying basic population genetic analyses in R. Methods in Ecology and Evolution, 5, 384–387.

[ece35685-bib-0002] Andersen, L. , Fog, K. , & Damgaard, C. (2004). Habitat fragmentation causes bottlenecks and inbreeding in the European tree frog (*Hyla arborea*). Proceedings of the Royal Society B: Biological Sciences, 271, 1293–1302. 10.1098/rspb.2004.2720 PMC1691722 15306354

[ece35685-bib-0003] Andrén, H. , & Andren, H. (1994). Effects of habitat fragmentation on birds and mammals in landscapes with different proportions of suitable habitat: A review. Oikos, 71, 355–366. 10.2307/3545823

[ece35685-bib-0004] Andrews, K. M. , Gibbons, J. W. , & Jochimsen, D. M. (2008). Ecological effects of roads on amphibians and reptiles: A literature review. Herpetological Conservation, 3, 121–143.

[ece35685-bib-0005] Baldwin, R. F. , Calhoun, A. J. K. , & de Maynadier, P. G. (2006a). Conservation planning for amphibian species with complex habitat requirements: A case study using movements and habitat selection of the wood frog *Rana sylvatica* . Journal of Herpetology, 40, 442–453. 10.1670/0022-1511(2006)40[442:CPFASW]2.0.CO;2

[ece35685-bib-0006] Baldwin, R. F. , Calhoun, A. J. K. , & de Maynadier, P. G. (2006b). The significance of hydroperiod and stand maturity for pool‐breeding amphibians in forested landscapes. Canadian Journal of Zoology, 84, 1604–1615. 10.1139/z06-146

[ece35685-bib-0007] Baldwin, R. F. , & de Maynadier, P. G. (2009). Assessing threats to pool‐breeding amphibian habitat in an urbanizing landscape. Biological Conservation, 142, 1628–1638. 10.1016/j.biocon.2009.02.039

[ece35685-bib-0008] Balkenhol, N. , Cushman, S. A. , Waits, L. P. , & Storfer, A. (2016). Current status, future opportunities, and remaining challenges in landscape genetics In Waits L., Storfer A., Cushman S. A., & Balkenhol N. (Eds.), Landscape genetics: Concepts, methods, applications (pp. 247–255).

[ece35685-bib-0009] Bascompte, J. , & Sole, R. V. (1996). Habitat fragmentation and extinction thresholds in spatially explicit models. The Journal of Animal Ecology, 65, 465–473. 10.2307/5781

[ece35685-bib-0010] Benjamini, Y. , & Hochberg, Y. (1995). Controlling the false discovery rate: A practical and powerful approach to multiple testing. Journal of the Royal Statistical Society: Series B (Methodological), 57, 289–300. 10.1111/j.2517-6161.1995.tb02031.x

[ece35685-bib-0011] Berven, K. A. , & Grudzien, T. A. (1990). Dispersal in the wood frog (*Rana sylvatica*): Implications for genetic population structure. Evolution, 44, 2047–2056. 28564421 10.1111/j.1558-5646.1990.tb04310.x

[ece35685-bib-0012] Blomquist, S. M. , & Hunter, M. L., Jr. (2010). A multi‐scale assessment of amphibian habitat selection: Wood frog response to timber harvesting. Ecoscience, 17, 251–264. 10.2980/17-3-3316

[ece35685-bib-0013] Borcard, D. , & Legendre, P. (2012). Is the Mantel correlogram powerful enough to be useful in ecological analysis? A simulation study. Ecology, 93, 1473–1481. 10.1890/11-1737.1 22834387

[ece35685-bib-0014] Bradbury, I. R. , & Bentzen, P. (2007). Non‐linear genetic isolation by distance: Implications for dispersal estimations in anadromous and marine fish populations. Marine Ecology Progress Series, 340, 245–257.

[ece35685-bib-0015] Burkhart, J. J. , Peterman, W. E. , Brocato, E. R. , Romine, K. M. , Willis, M. M. S. , Ousterhout, B. H. , … Eggert, L. S. (2017). The influence of breeding phenology on the genetic structure of four pond‐breeding salamanders. Ecology and Evolution, 7(13), 4670–4681. 10.1002/ece3.3060 28690797 PMC5496555

[ece35685-bib-0016] Burnham, K. P. , & Anderson, D. (2002). Model selection and multimodel inference: A practical information‐theoretic approach. Berlin, Germany: Springer Science and Business Media.

[ece35685-bib-0017] Calcagno, V. , & de Mazancourt, C. (2010). glmulti: An R package for easy automated model selection with (generalized) linear models. Journal of Statistical Software, 34, 1–29.

[ece35685-bib-0018] Cheptou, P.‐O. , Hargreaves, A. L. , Bonte, D. , & Jacquemyn, H. (2017). Adaptation to fragmentation: Evolutionary dynamics driven by human influences. Philosophical Transactions of the Royal Society B: Biological Sciences, 372, 20160037 10.1098/rstb.2016.0037 PMC5182433 27920382

[ece35685-bib-0019] Clark, P. J. , Reed, J. M. , Tavernia, B. G. , Windmiller, B. S. , & Regosin, J. V. (2008). Urbanization effects on spotted salamander and wood frog presence and abundance. Urban Herpetology, 3, 67–75.

[ece35685-bib-0020] Cline, B. B. , & Hunter, M. L. (2014). Different open‐canopy vegetation types affect matrix permeability for a dispersing forest amphibian. Journal of Applied Ecology, 51, 319–329. 10.1111/1365-2664.12197

[ece35685-bib-0021] Cline, B. B. , & Hunter, M. L. (2016). Movement in the matrix: Substrates and distance‐to‐forest edge affect postmetamorphic movements of a forest amphibian. Ecosphere, 7, 1–23. 10.1002/ecs2.1202

[ece35685-bib-0022] Combs, M. , Puckett, E. E. , Richardson, J. , Mims, D. , & Munshi‐South, J. (2017). Spatial population genomics of the brown rat (*Rattus norvegicus*) in New York City. Molecular Ecology, 12, 3218–3221. 10.1111/mec.14437 29165929

[ece35685-bib-0023] Cornuet, J. M. , & Luikart, G. (1996). Description and power analysis of two tests for detecting recent population bottlenecks from allele frequency data. Genetics, 144, 2001–2014. 8978083 10.1093/genetics/144.4.2001 PMC1207747

[ece35685-bib-0024] Cosentino, B. J. , Phillips, C. A. , Schooley, R. L. , Lowe, W. H. , & Douglas, M. R. (2012). Linking extinction‐colonization dynamics to genetic structure in a salamander metapopulation. Proceedings of the Royal Society B: Biological Sciences, 279, 1575–1582. 10.1098/rspb.2011.1880 PMC3282340 22113029

[ece35685-bib-0025] Coster, S. S. , Babbitt, K. J. , Cooper, A. , & Kovach, A. I. (2015). Limited influence of local and landscape factors on finescale gene flow in two pond‐breeding amphibians. Molecular Ecology, 24, 742–758. 10.1111/mec.13062 25580642

[ece35685-bib-0026] Coster, S. S. , Babbitt, K. J. , & Kovach, A. I. (2015). High genetic connectivity in wood frogs (*Lithobates sylvaticus*) and spotted salamanders (*Ambystoma maculatum*) in a commercial forest. Herpetological Conservation and Biology, 10, 64–89.

[ece35685-bib-0027] Cox, K. , Maes, J. , van Calster, H. , & Mergeay, J. (2017). Effect of the landscape matrix on gene flow in a coastal amphibian metapopulation. Conservation Genetics, 18(6), 1359–1375. 10.1007/s10592-017-0985-z

[ece35685-bib-0028] Crawford, J. A. , Peterman, W. E. , Kuhns, A. R. , & Eggert, L. S. (2016). Altered functional connectivity and genetic diversity of a threatened salamander in an agroecosystem. Landscape Ecology, 31, 2231–2244. 10.1007/s10980-016-0394-6

[ece35685-bib-0029] Crooks, K. R. , Burdett, C. L. , Theobald, D. M. , King, S. R. B. , Di Marco, M. , Rondinini, C. , & Boitani, L. (2017). Quantification of habitat fragmentation reveals extinction risk in terrestrial mammals. Proceedings of the National Academy of Sciences of the United States of America, 114, 7635–7640. 10.1073/pnas.1705769114 28673992 PMC5530695

[ece35685-bib-0030] Crosby, M. K. A. , Licht, L. E. , & Fu, J. (2008). The effect of habitat fragmentation on finescale population structure of wood frogs (*Rana sylvatica*). Conservation Genetics, 10, 1707–1718. 10.1007/s10592-008-9772-1

[ece35685-bib-0031] Cushman, S. A. , & Landguth, E. L. (2010). Scale dependent inference in landscape genetics. Landscape Ecology, 25, 967–979. 10.1007/s10980-010-9467-0 20618896

[ece35685-bib-0032] De Roissart, A. , Wybouw, N. , Renault, D. , Van Leeuwen, T. , & Bonte, D. (2016). Life‐history evolution in response to changes in metapopulation structure in an arthropod herbivore. Functional Ecology, 30, 1408–1417.

[ece35685-bib-0033] Di Rienzo, A. , Peterson, A. C. , Garzat, J. C. , Valdes, A. M. , Slatkin, M. , & Freimer, N. B. (1994). Mutational processes of simple‐sequence repeat loci in human populations. Genetics, 91, 3166–3170. 10.1073/pnas.91.8.3166 PMC43536 8159720

[ece35685-bib-0034] Dudaniec, R. Y. , Spear, S. F. , Richardson, J. S. , & Storfer, A. (2012). Current and historical drivers of landscape genetic structure differ in core and peripheral salamander populations. PLoS ONE, 7, e36769 10.1371/journal.pone.0036769 22590604 PMC3349670

[ece35685-bib-0035] Durand, E. , Jay, F. , Gaggiotti, O. E. , & François, O. (2009). Spatial inference of admixture proportions and secondary contact zones. Molecular Biology and Evolution, 26, 1963–1973. 10.1093/molbev/msp106 19461114

[ece35685-bib-0036] Eckert, C. G. , Samis, K. E. , & Lougheed, S. C. (2008). Genetic variation across species' geographical ranges: The central‐marginal hypothesis and beyond. Molecular Ecology, 17, 1170–1188. 10.1111/j.1365-294X.2007.03659.x 18302683

[ece35685-bib-0037] Epps, C. W. , & Keyghobadi, N. (2015). Landscape genetics in a changing world: Disentangling historical and contemporary influences and inferring change. Molecular Ecology, 24, 6021–6040. 10.1111/mec.13454 26547281

[ece35685-bib-0038] ESRI (2013). ArcGIS 10.2 for Desktop. Redlands, CA: ESRI, Inc.

[ece35685-bib-0039] Evans, J. S. (2017). _spatialEco_. R package version 0.0.1‐7. Retrieved from https://CRAN.R-project.org/package=spatialEco

[ece35685-bib-0040] Excoffier, L. , & Lischer, H. E. L. (2010). Arlequin suite ver 3.5: A new series of programs to perform population genetics analyses under Linux and Windows. Molecular Ecology Resources, 10, 564–567. 21565059 10.1111/j.1755-0998.2010.02847.x

[ece35685-bib-0041] Ferris, C. R. (1979). Effects of Interstate 95 on breeding birds in northern Maine. Journal of Wildlife Management, 43, 421–427. 10.2307/3800351

[ece35685-bib-0042] Flageole, S. , & Leclair, R., Jr. (1992). Étude démographique d'une population de salamandres (*Ambystoma maculatum*) à l'aide de la méthode squeletto‐chronologique. Canadian Journal of Zoology, 70, 740–749.

[ece35685-bib-0043] Fox, J. , & Weisberg, S. (2011). An R companion to applied regression (2nd ed.). Thousand Oaks, CA: Sage.

[ece35685-bib-0044] Frankham, R. (2015). Genetic rescue of small inbred populations: Meta‐analysis reveals large and consistent benefits of gene flow. Molecular Ecology, 24(11), 2610–2618. 10.1111/mec.13139 25740414

[ece35685-bib-0045] Fronhofer, E. A. , & Altermatt, F. (2017). Classical metapopulation dynamics and eco‐evolutionary feedbacks in dendritic networks. Ecography, 40, 1455–1466. 10.1111/ecog.02761

[ece35685-bib-0046] Gabrielsen, C. G. , Kovach, A. I. , Babbitt, K. J. , & McDowell, W. H. (2013). Limited effects of suburbanization on the genetic structure of an abundant vernal pool-breeding amphibian. Conservation Genetics, 14(5), 1083–1097.

[ece35685-bib-0047] Galpern, P. , & Manseau, M. (2013). Finding the functional grain: Comparing methods for scaling resistance surfaces. Landscape Ecology, 28(7), 1269–1281. 10.1007/s10980-013-9873-1

[ece35685-bib-0048] Garcia, V. O. S. , Ivy, C. , & Fu, J. (2017). Syntopic frogs reveal different patterns of interaction with the landscape: A comparative landscape genetic study of *Pelophylax nigromaculatus* and *Fejervarya limnocharis* from central China. Ecology and Evolution, 7, 9294–9306. 29187969 10.1002/ece3.3459 PMC5696414

[ece35685-bib-0049] Goldberg, C. S. , & Waits, L. P. (2010). Quantification and reduction of bias from sampling larvae to infer population and landscape genetic structure. Molecular Ecology Resources, 10, 304–313. 10.1111/j.1755-0998.2009.02755.x 21565025

[ece35685-bib-0050] Goudet, J. , & Jombart, T. (2015). hierfstat: Estimation and tests of hierarchical F‐statistics. R package version 0.04‐22. Retrieved from https://CRAN.R-project.org/package=hierfstat

[ece35685-bib-0051] Graham, L. J. , Haines‐Young, R. H. , & Field, R. (2017). Metapopulation modelling of long‐term urban habitat‐loss scenarios. Landscape Ecology, 32, 989–1003. 10.1007/s10980-017-0504-0 PMC7010366 32103856

[ece35685-bib-0052] Green, A. W. , Hooten, M. B. , Grant, E. H. C. , & Bailey, L. L. (2013). Evaluating breeding and metamorph occupancy and vernal pool management effects for wood frogs using a hierarchical model. Journal of Applied Ecology, 50, 1116–1123. 10.1111/1365-2664.12121

[ece35685-bib-0053] Grilli, J. , Barabás, G. , & Allesina, S. (2015). Metapopulation persistence in random fragmented landscapes. PLoS Computational Biology, 11, 1–13. 10.1371/journal.pcbi.1004251 PMC4439033 25993004

[ece35685-bib-0054] Groff, L. A. , Calhoun, A. J. K. , & Loftin, C. S. (2017). Amphibian terrestrial habitat selection and movement patterns vary with annual life-history period. Canadian Journal of Zoology, 95(6), 433–442.

[ece35685-bib-0055] Haddad, N. M. , Brudvig, L. A. , Clobert, J. , Davies, K. F. , Gonzalez, A. , Holt, R. D. , … Townshend, J. R. (2015). Habitat fragmentation and its lasting impact on Earth's ecosystems. Science Advances, 1, e1500052 10.1126/sciadv.1500052 26601154 PMC4643828

[ece35685-bib-0056] Hale, J. M. , Heard, G. W. , Smith, K. L. , Parris, K. M. , Austin, J. J. , Kearney, M. , & Melville, J. (2013). Structure and fragmentation of growling grass frog metapopulations. Conservation Genetics, 14, 313–322. 10.1007/s10592-012-0428-9

[ece35685-bib-0057] Hamer, A. J. , Langton, T. E. S. , & Lesbarreres, D. (2015). Making the safe leap forward: mitigating road impacts on amphibians In van der Ree R., Smith D. J., & Grilo C. (Eds.), Handbook of road ecology (pp. 261–270). Hoboken, NJ: John Wiley & Sons.

[ece35685-bib-0058] Hanski, I. (1998). Metapopulation dynamics. Trends in Ecology & Evolution, 396, 41–49. 10.1038/23876 21227332

[ece35685-bib-0059] Hanski, I. , & Gilpin, M. (1991). Metapopulation dynamics: Brief history and conceptual domain. Biological Journal of the Linnean Society, 42, 3–16. 10.1111/j.1095-8312.1991.tb00548.x

[ece35685-bib-0060] Harper, E. B. , Rittenhouse, T. A. G. , & Semlitsch, R. D. (2008). Demographic consequences of terrestrial habitat loss for pool‐breeding amphibians: Predicting extinction risks associated with inadequate size of buffer zones. Conservation Biology, 22, 1205–1215. 10.1111/j.1523-1739.2008.01015.x 18717698

[ece35685-bib-0061] Heard, G. W. , McCarthy, M. A. , Scroggie, M. P. , Baumgartner, J. B. , & Parris, K. M. (2013). A Bayesian model of metapopulation viability, with application to an endangered amphibian. Diversity and Distributions, 19, 555–566. 10.1111/ddi.12052

[ece35685-bib-0062] Hijmans, R. J. (2017). geosphere: Spherical Trigonometry. R package version 1.5‐7. Retrieved from https://CRAN.R-project.org/package=geosphere

[ece35685-bib-0063] Holderegger, R. , & Di Giulio, M. (2010). The genetic effects of roads: A review of empirical evidence. Basic and Applied Ecology, 11(6), 522–531.

[ece35685-bib-0064] Homan, R. N. , Windmiller, B. S. , & Reed, J. M. (2004). Critical thresholds associated with habitat loss for two vernal pool‐breeding amphibians. Ecological Applications, 14, 1547–1553. 10.1890/03-5125

[ece35685-bib-0065] Homer, C. , Dewitz, J. , Yang, L. , Jin, S. , Danielson, P. , Xian, G. , … Megown, K. (2015). Completion of the 2011 National Land Cover Database for the conterminous United States – Representing a decade of land cover change information. Photogrammetric Engineering & Remote Sensing, 81, 345–354.

[ece35685-bib-0066] Hutchison, D. W. , & Templeton, A. R. (1999). Correlation of pairwise genetic and geographic distance measures: Inferring the relative influences of gene flow and drift on the distribution of genetic variability. Evolution, 53, 1898 10.2307/2640449 28565459

[ece35685-bib-0067] Jenkins, D. G. , Carey, M. , Czerniewska, J. , Fletcher, J. , Hether, T. , Jones, A. , … Tursi, R. (2010). A meta‐analysis of isolation by distance: Relic or reference standard for landscape genetics? Ecography, 33, 315–320. 10.1111/j.1600-0587.2010.06285.x

[ece35685-bib-0068] Johnson, P. , Pitcher, J. , Prendergast, J. , Jackman, G. , Moulton, N. , Bistrias, B. , & Beecher, J. (2011). RailRouteSys. Augusta, ME: Maine Department of Transportation.

[ece35685-bib-0069] Jones, O. R. , & Wang, J. (2010). COLONY: A program for parentage and sibship inference from multilocus genotype data. Molecular Ecology Resources, 10, 551–555. 10.1111/j.1755-0998.2009.02787.x 21565056

[ece35685-bib-0070] Kenney, J. , Allendorf, F. W. , Mcdougal, C. , & Smith, J. L. (2014). How much gene flow is needed to avoid inbreeding depression in wild tiger populations? Proceedings of the Royal Society B: Biological Sciences, 281(1789), 20133337 10.1098/rspb.2013.3337 PMC4100497 24990671

[ece35685-bib-0071] Kimura, M. , & Weiss, G. H. (1964). The stepping stone model of population structure and the decrease of genetic correlation with distance. Genetics, 49, 561–576. 17248204 10.1093/genetics/49.4.561 PMC1210594

[ece35685-bib-0072] Koen, E. L. , Bowman, J. , Garroway, C. J. , & Wilson, P. J. (2013). The sensitivity of genetic connectivity measures to unsampled and under‐sampled sites. PLoS ONE, 8(2), e56204. 23409155 10.1371/journal.pone.0056204 PMC3568052

[ece35685-bib-0073] Koizumi, I. , Yamamoto, S. , & Maekawa, K. (2006). Decomposed pairwise regression analysis of genetic and geographic distances reveals a metapopulation structure of stream‐dwelling Dolly Varden charr. Molecular Ecology, 15, 3175–3189. 10.1111/j.1365-294X.2006.03019.x 16968263

[ece35685-bib-0074] Landguth, E. L. , Cushman, S. A. , Schwartz, M. K. , McKelvey, K. S. , Murphy, M. , & Luikart, G. (2010). Quantifying the lag time to detect barriers in landscape genetics. Molecular Ecology, 19, 4179–4191. 10.1111/j.1365-294X.2010.04808.x 20819159

[ece35685-bib-0075] Legendre, P. , Lapointe, F. J. , & Casgrain, P. (1994). Modeling brain evolution from behavior: A permutational regression approach. Evolution, 48, 1487–1499. 10.1111/j.1558-5646.1994.tb02191.x 28568410

[ece35685-bib-0076] Legendre, P. , & Legendre, F. J. (2012). Numerical ecology (3rd ed.). Amsterdam, The Netherlands: Elsevier.

[ece35685-bib-0077] Leonard, P. B. , Duffy, E. B. , Baldwin, R. F. , McRae, B. H. , Shah, V. B. , & Mohapatra, T. K. (2017). Gflow: Software for modelling circuit theory‐based connectivity at any scale. Methods in Ecology and Evolution, 8, 519–526.

[ece35685-bib-0078] Levins, R. (1969). Some demographic and genetic consequences of environmental heterogeneity for biological control. American Entomologist, 15, 237–240.

[ece35685-bib-0079] Lopez, S. , Rousset, F. , Shaw, F. H. , Shaw, R. G. , & Ronce, O. (2009). Joint effects of inbreeding and local adaptation on the evolution of genetic load after fragmentation. Conservation Biology, 23, 1618–1627. 10.1111/j.1523-1739.2009.01326.x 19775278

[ece35685-bib-0080] Madison, D. M. (1997). The emigration of radio‐implanted spotted salamanders, *Ambystoma maculatum* . Journal of Herpetology, 31, 542–551. 10.2307/1565607

[ece35685-bib-0081] Manel, S. , & Holderegger, R. (2013). Ten years of landscape genetics. Trends in Ecology and Evolution, 28, 614–621. 10.1016/j.tree.2013.05.012 23769416

[ece35685-bib-0082] Mantel, N. (1967). The detection of disease clustering and a generalized regression approach. Cancer Research, 27, 209–220. 6018555

[ece35685-bib-0083] Maruyama, T. , & Fuerst, P. A. (1985). Population bottlenecks and nonequilibrium models in population genetics. II. Number of alleles in a small population that was formed by a recent bottleneck. Genetics, 111, 675–689. 4054612 10.1093/genetics/111.3.675 PMC1202664

[ece35685-bib-0084] McCallum, M. L. (2007). Amphibian decline or extinction? Current declines dwarf background extinction rate. Journal of Herpetology, 41, 483–491. 10.1670/0022-1511(2007)41[483:ADOECD]2.0.CO;2

[ece35685-bib-0085] McKee, A. M. , Maerz, J. C. , Smith, L. L. , & Glenn, T. C. (2017). Habitat predictors of genetic diversity for two sympatric wetland‐breeding amphibian species. Ecology and Evolution, 7(16), 6271–6283. 10.1002/ece3.3203 28861231 PMC5574763

[ece35685-bib-0086] Meirmans, P. G. , & Hedrick, P. W. (2011). Assessing population structure: FST and related measures. Molecular Ecology Resources, 11, 5–18. 10.1111/j.1755-0998.2010.02927.x 21429096

[ece35685-bib-0087] Millsap, B. A. (2018). Demography and metapopulation dynamics of an urban Cooper's Hawk subpopulation. The Condor, 120, 63–80. 10.1650/CONDOR-17-124.1

[ece35685-bib-0088] Moyle, L. C. (2006). Correlates of genetic differentiation and isolation by distance in 17 congeneric Silene species. Molecular Ecology, 15(4), 1067–1081. 16599967 10.1111/j.1365-294X.2006.02840.x

[ece35685-bib-0089] Munshi‐South, J. , Zolnik, C. P. , & Harris, S. E. (2016). Population genomics of the Anthropocene: Urbanization is negatively associated with genome‐wide variation in white‐footed mouse populations. Evolutionary Applications, 9, 546–564. 10.1111/eva.12357 27099621 PMC4831458

[ece35685-bib-0090] Nei, M. (1973). Analysis of gene diversity in subdivided populations. Proceedings of the National Academy of Sciences of the United States of America, 70, 3321–3323. 10.1073/pnas.70.12.3321 4519626 PMC427228

[ece35685-bib-0091] Nei, M. , & Chesser, R. K. (1983). Estimation of fixation indices and gene diversities. Annals of Human Genetics, 47, 253–259. 10.1111/j.1469-1809.1983.tb00993.x 6614868

[ece35685-bib-0092] Oden, N. L. , & Sokal, R. R. (1986). Directional autocorrelation: An extension of spatial correlograms to two dimensions. Systematic Zoology, 35, 608–617. 10.2307/2413120

[ece35685-bib-0093] Oksanen, J. , Blanchet, F. G. , Friendly, M. , Kindt, R. , Legendre, P. , Mcglinn, D. , … Wagner, H. (2017). The vegan package: Community ecology package. Vienna, Austria: R Foundation for Statistical Computing.

[ece35685-bib-0094] Ortego, J. , Aguirre, M. P. , Noguerales, V. , & Cordero, P. J. (2015). Consequences of extensive habitat fragmentation in landscape‐level patterns of genetic diversity and structure in the Mediterranean esparto grasshopper. Evolutionary Applications, 8, 621–632. 10.1111/eva.12273 26136826 PMC4479516

[ece35685-bib-0095] Ousterhout, B. H. , & Burkhart, J. J. (2017). Moving beyond the plane: measuring 3D home ranges of juvenile salamanders with passive integrated transponder (PIT) tags. Behavioral Ecology and Sociobiology, 71(4).

[ece35685-bib-0096] Padilla, B. J. , & Rodewald, A. D. (2015). Avian metapopulation dynamics in a fragmented urbanizing landscape. Urban Ecosystems, 18, 239–250. 10.1007/s11252-014-0390-z

[ece35685-bib-0097] Pavlova, A. , Beheregaray, L. B. , Coleman, R. , Gilligan, D. , Harrisson, K. A. , Ingram, B. A. , … Sunnucks, P. (2017). Severe consequences of habitat fragmentation on genetic diversity of an endangered Australian freshwater fish: A call for assisted gene flow. Evolutionary Applications, 10, 531–550. 10.1111/eva.12484 28616062 PMC5469170

[ece35685-bib-0098] Peterman, W. E. , Anderson, T. L. , Ousterhout, B. H. , Drake, D. L. , Semlitsch, R. D. , & Eggert, L. S. (2015). Differential dispersal shapes population structure and patterns of genetic differentiation in two sympatric pond breeding salamanders. Conservation Genetics, 16, 59–69. 10.1007/s10592-014-0640-x

[ece35685-bib-0099] Peterman, W. , Brocato, E. R. , Semlitsch, R. D. , & Eggert, L. S. (2016). Reducing bias in population and landscape genetic inferences: The effects of sampling related individuals and multiple life stages. PeerJ, 4, e1813 10.7717/peerj.1813 26989639 PMC4793335

[ece35685-bib-0100] Peterman, W. E. , Feist, S. M. , Semlitsch, R. D. , & Eggert, L. S. (2013). Conservation and management of peripheral populations: Spatial and temporal influences on the genetic structure of wood frog (*Rana sylvatica*) populations. Biological Conservation, 158, 351–358. 10.1016/j.biocon.2012.07.028

[ece35685-bib-0101] Petkova, D. , Novembre, J. , & Stephens, M. (2016). Visualizing spatial population structure with estimated effective migration surfaces. Nature Genetics, 48, 94–100. 10.1038/ng.3464 26642242 PMC4696895

[ece35685-bib-0102] Phillipsen, I. C. , Kirk, E. H. , Bogan, M. T. , Mims, M. C. , Olden, J. D. , & Lytle, D. A. (2015). Dispersal ability and habitat requirements determine landscape-level genetic patterns in desert aquatic insects. Molecular Ecology, 24(1), 54–69. 25402260 10.1111/mec.13003

[ece35685-bib-0103] Piry, S. , Luikart, G. , & Cornuet, J. M. (1999). BOTTLENECK: A computer program for detecting recent reductions in the effective population size using allele frequency data. Journal of Heredity, 90, 502–503.

[ece35685-bib-0104] Pittman, S. E. , Osbourn, M. S. , & Semlitsch, R. D. (2014). Movement ecology of amphibians: A missing component for understanding population declines. Biological Conservation, 169, 44–53. 10.1016/j.biocon.2013.10.020

[ece35685-bib-0105] Price, S. J. , Dorcas, M. E. , Gallant, A. L. , Klaver, R. W. , & Willson, J. D. (2006). Three decades of urbanization: Estimating the impact of land‐cover change on stream salamander populations. Biological Conservation, 133, 436–441. 10.1016/j.biocon.2006.07.005

[ece35685-bib-0106] Purrenhage, J. L. , Niewiarowski, P. H. , & Moore, F. B. G. (2009). Population structure of spotted salamanders (*Ambystoma maculatum*) in a fragmented landscape. Molecular Ecology, 18, 235–247. 19192178 10.1111/j.1365-294X.2008.04024.x

[ece35685-bib-0107] R Core Team (2016). R: A language and environment for statistical computing. Vienna, Austria: R Foundation for Statistical Computing.

[ece35685-bib-0108] Raymond, M. , & Rousset, F. (1995). GENEPOP (version 1.2): Population genetics software for exact tests and ecumenicism. Journal of Heredity, 86, 248–249.

[ece35685-bib-0109] Reigada, C. , Schreiber, S. J. , Altermatt, F. , & Holyoak, M. (2015). Metapopulation dynamics on ephemeral patches. The American Naturalist, 185, 183–195. 10.1086/679502 25616138

[ece35685-bib-0110] Richardson, J. L. (2012). Divergent landscape effects on population connectivity in two co‐occurring amphibian species. Molecular Ecology, 21, 4437–4451. 10.1111/j.1365-294X.2012.05708.x 22891686

[ece35685-bib-0111] Richardson, J. L. , Brady, S. P. , Wang, I. J. , & Spear, S. F. (2016). Navigating the pitfalls and promise of landscape genetics. Molecular Ecology, 25, 849–863. 10.1111/mec.13527 26756865

[ece35685-bib-0112] Riley, S. J. , DeGloria, S. D. , & Elliot, R. (1999). A terrain ruggedness index that quantifies topographic heterogeneity. Intermountain Journal of Sciences, 5, 23–27.

[ece35685-bib-0113] Rodríguez‐Ramilo, S. T. , & Wang, J. (2012). The effect of close relatives on unsupervised Bayesian clustering algorithms in population genetic structure analysis. Molecular Ecology Resources, 12, 873–884. 10.1111/j.1755-0998.2012.03156.x 22639868

[ece35685-bib-0114] Rousset, F. (2000). Genetic differentiation between individuals. Journal of Evolutionary Biology, 13, 58–62. 10.1046/j.1420-9101.2000.00137.x

[ece35685-bib-0115] Rousset, F. (2004). Genetic structure and selection in subdivided populations. Princeton, NJ: Princeton University Press.

[ece35685-bib-0116] Rousset, F. (2008). GENEPOP'007: A complete re‐implementation of the GENEPOP software for Windows and Linux. Molecular Ecology Resources, 8, 103–106. 10.1111/j.1471-8286.2007.01931.x 21585727

[ece35685-bib-0117] Sagor, E. S. , Ouellet, M. , Barten, E. , & Green, D. M. (1998). Skeletochronology and geographic variation in age structure in the wood frog, *Rana sylvatica* . Journal of Herpetology, 32, 469.

[ece35685-bib-0118] Schmidt, B. R. , & Zumbach, S. (2008). Amphibian road mortality and how to prevent it: A review. Herpetological Conservation, 3, 157–167.

[ece35685-bib-0119] Schnell, J. K. , Harris, G. M. , Pimm, S. L. , & Russell, G. J. (2013). Estimating extinction risk with metapopulation models of large‐scale fragmentation. Conservation Biology, 27, 520–530. 10.1111/cobi.12047 23551595

[ece35685-bib-0120] Semlitsch, R. D. (1998). Biological delineation of terrestrial buffer zones for pond‐breeding salamanders. Conservation Biology, 12, 1113–1119. 10.1046/j.1523-1739.1998.97274.x

[ece35685-bib-0121] Semlitsch, R. D. (2003). Conservation of pond‐breeding amphibians In Semlitsch R. D. (Ed.), Amphibian conservation (pp. 8–23). Washington, DC: Smithsonian Institute Press.

[ece35685-bib-0122] Semlitsch, R. D. (2008). Differentiating migration and dispersal processes for pond‐breeding amphibians. Journal of Wildlife Management, 72, 260–267. 10.2193/2007-082

[ece35685-bib-0123] Semlitsch, R. D. , & Jensen, J. B. (2001). Core habitat, not buffer zone. National Wetlands Newsletter, 23, 4–6.

[ece35685-bib-0124] Seto, K. C. , Sánchez‐Rodríguez, R. , & Fragkias, M. (2010). The new geography of contemporary urbanization and the environment. Annual Review of Environment and Resources, 35, 167–194. 10.1146/annurev-environ-100809-125336

[ece35685-bib-0125] Sharma, S. , Dutta, T. , Maldonado, J. E. , Wood, T. C. , Panwar, H. S. , & Seidensticker, J. (2013). Forest corridors maintain historical gene flow in a tiger metapopulation in the highlands of central India. Proceedings of the Royal Society B: Biological Sciences, 280, 20131506 10.1098/rspb.2013.1506 PMC3735263 23902910

[ece35685-bib-0126] Short Bull, R. A. , Cushman, S. A. , Mace, R. , Chilton, T. , Kendall, K. C. , Landguth, E. L. , … Luikart, G. (2011). Why replication is important in landscape genetics: American black bear in the Rocky Mountains. Molecular Ecology, 20, 1092–1107. 10.1111/j.1365-294X.2010.04944.x 21261764

[ece35685-bib-0127] Slatkin, M. (1995). A measure of population subdivision based on microsatellite allele frequencies. Genetics, 139, 457–462. 7705646 10.1093/genetics/139.1.457 PMC1206343

[ece35685-bib-0128] Squire, T. , & Newman, R. A. (2002). Fine‐scale population structure in the wood frog (*Rana sylvatica*) in a northern woodland. Herpetologica, 58, 119–130. 10.1655/0018-0831(2002)058[0119:FPSITW]2.0.CO;2 11380868

[ece35685-bib-0129] Theobald, D. M. (2010). Estimating natural landscape changes from 1992 to 2030 in the conterminous US. Landscape Ecology, 25, 999–1011. 10.1007/s10980-010-9484-z

[ece35685-bib-0130] Titus, V. R. , Bell, R. C. , Becker, C. G. , & Zamudio, K. R. (2014). Connectivity and gene flow among Eastern Tiger Salamander (Ambystoma tigrinum) populations in highly modified anthropogenic landscapes. Conservation Genetics, 15(6), 1447–1462.

[ece35685-bib-0131] Trumbo, D. R. , Spear, S. F. , Baumsteiger, J. , & Storfer, A. (2013). Rangewide landscape genetics of an endemic Pacific northwestern salamander. Molecular Ecology, 22(5), 1250–1266. 23293948 10.1111/mec.12168

[ece35685-bib-0132] UNDESA (2012). World urbanization prospects: The 2011 revision. United Nations Department of Economics and Social Affairs. New York, NY: Population Division.

[ece35685-bib-0133] UNDESA (2015). World urbanization prospects: The 2014 revision. United Nations Department of Economics and Social Affairs. New York, NY: Population Division.

[ece35685-bib-0134] van Strien, M. J. , Holderegger, R. , & Van Heck, H. J. (2015). Isolation‐by‐distance in landscapes: Considerations for landscape genetics. Heredity, 114, 27–37. 10.1038/hdy.2014.62 25052412 PMC4815601

[ece35685-bib-0135] Vasconcelos, D. , & Calhoun, A. J. K. (2004). Movement patterns of adult and juvenile *Rana sylvatica* (LeConte) and *Ambystoma maculatum* (Shaw) in three restored seasonal pools in Maine. Journal of Herpetology, 38, 551–561. 10.1670/157-03A

[ece35685-bib-0136] Wagner, H. H. , Holderegger, R. , Werth, S. , Gugerli, F. , Hoebee, S. E. , & Scheidegger, C. (2005). Variogram analysis of the spatial genetic structure of continuous populations using multilocus microsatellite data. Genetics, 169, 1739–1752. 10.1534/genetics.104.036038 15654102 PMC1449570

[ece35685-bib-0137] Wang, I. (2013). Examining the full effects of landscape heterogeneity on spatial genetic variation: A multiple matrix regression approach for quantifying geographic and ecological isolation. Evolution, 67, 3403–3411. 10.1111/evo.12134 24299396

[ece35685-bib-0138] Wang, J. (2004). Sibship reconstruction from genetic data with typing errors. Genetics, 166, 1963–1979. 10.1534/genetics.166.4.1963 15126412 PMC1470831

[ece35685-bib-0139] Wang, J. (2018). Effects of sampling close relatives on some elementary population genetics analyses. Molecular Ecology Resources, 18, 41–54. 10.1111/1755-0998.12708 28776944

[ece35685-bib-0140] Waples, R. S. , & Anderson, E. C. (2017). Purging putative siblings from population genetic data sets: A cautionary view. Molecular Ecology, 26(5), 1211–1224. 10.1111/mec.14022 28099771

[ece35685-bib-0141] Whiteley, A. R. , McGarigal, K. , & Schwartz, M. K. (2014). Pronounced differences in genetic structure despite overall ecological similarity for two Ambystoma salamanders in the same landscape. Conservation Genetics, 15, 573–591. 10.1007/s10592-014-0562-7

[ece35685-bib-0142] Wilcox, B. A. , & Murphy, D. D. (1985). Conservation strategy: The effects of fragmentation on extinction. The American Naturalist, 125, 879–887. 10.1086/284386

[ece35685-bib-0143] Winter, D. J. (2012). MMOD: An R library for the calculation of population differentiation statistics. Molecular Ecology Resources, 12, 1158–1160. 22883857 10.1111/j.1755-0998.2012.03174.x

[ece35685-bib-0144] Youngquist, M. B. , Inoue, K. , Berg, D. J. , & Boone, M. D. (2017). Effects of land use on population presence and genetic structure of an amphibian in an agricultural landscape. Landscape Ecology, 32(1), 147–162.

[ece35685-bib-0145] Zamudio, K. R. , & Wieczorek, A. M. (2007). Fine‐scale spatial genetic structure and dispersal among spotted salamander (*Ambystoma maculatum*) breeding populations. Molecular Ecology, 16, 257–274. 10.1111/j.1365-294X.2006.03139.x 17217343

[ece35685-bib-0146] Zeller, K. A. , McGarigal, K. , & Whiteley, A. R. (2012). Estimating landscape resistance to movement: a review. Landscape Ecology, 27(6), 777–797.

[ece35685-bib-0147] Zellmer, A. J. , & Knowles, L. L. (2009). Disentangling the effects of historic vs. contemporary landscape structure on population genetic divergence. Molecular Ecology, 18, 3593–3602. 19674302 10.1111/j.1365-294X.2009.04305.x

