# Supplementary material for: Landscape genetics reveals unique and shared effects of urbanization for two sympatric pool‐breeding amphibians
Source: Ecol Evol. 2019 Oct 1;9(20):11799–823. doi: 10.1002/ece3.5685 (PMC6822048; doi:10.1002/ece3.5685)
Supplement: Supplementary file 1 [file ECE3-9-11799-s001.docx]

APPENDIX A

**Microsatellite genotyping methods**

Polymerase chain reactions (PCRs) were conducted in 10 μL volumes containing 1.2 μL of template DNA, 2 μL of 5x polymerase buffer, 0.4 μL of forward and reverse primers for each of five loci contained in each multiplex (Table S1), 1.2 μL of 2.5 mmol*L^-1^ MgCl_2_, 0.2 mmol*L^-1^ dNTPs, and sterile water. Loci were assigned to one of two five locus multiplexes per species and fluorescently labelled with either 6-FAM, VIC, NED, or PET dyes (Applied Biosystems, Foster City, CA, USA; Table S1).

PCR conditions for spotted salamanders began with denaturing at 94°C for four minutes, followed by 30 cycles of 94°C for one minute, 58°C for one minute, and 72°C for one minute and 30 seconds. Finally, an extension period of five minutes at 72°C was performed. Wood frogs PCRs began with denaturing at 94°C for four minutes, followed by 26 cycles of 94°C for 45 seconds, 58°C for one minute, and 72°C for 1 minute before a final five minute extension period at 72°C.

Table A1. List of 10 spotted salamander and wood frog microsatellite loci used for analyses, including their multiplex arrangement, assigned fluorescence dye, and original citation.

| Species | Locus | Multiplex | Dye | Reference |
| --- | --- | --- | --- | --- |
| Spotted salamander | AmaD321 | 1 | PET | Julian et al. 2003a |
| Spotted salamander | AmaD184 | 1 | 6-FAM | Julian et al. 2003a |
| Spotted salamander | AmaD95 | 1 | NED | Julian et al. 2003a |
| Spotted salamander | AjeD23 | 1 | 6-FAM | Julian et al. 2003b |
| Spotted salamander | AmaD99 | 1 | VIC | Julian et al. 2003a |
| Spotted salamander | AmaD328 | 2 | NED | Julian et al. 2003a |
| Spotted salamander | AmaC40 | 2 | NED | Julian et al. 2003a |
| Spotted salamander | AmaD287 | 2 | 6-FAM | Julian et al. 2003a |
| Spotted salamander | AmaD49 | 2 | PET | Julian et al. 2003a |
| Spotted salamander | AmaD315 | 2 | VIC | Julian et al. 2003a |
| Wood frog | RsyC52 | 3 | 6-FAM | Julian and King 2003 |
| Wood frog | RsyD32 | 3 | VIC | Julian and King 2003 |
| Wood frog | RsyD40 | 3 | NED | Julian and King 2003 |
| Wood frog | RsyC83 | 3 | PET | Julian and King 2003 |
| Wood frog | RsyC23 | 3 | PET | Julian and King 2003 |
| Wood frog | RsyC11 | 4 | 6-FAM | Julian and King 2003 |
| Wood frog | RsyD88 | 4 | VIC | Julian and King 2003 |
| Wood frog | RsyD77 | 4 | NED | Julian and King 2003 |
| Wood frog | RsyC41 | 4 | PET | Julian and King 2003 |
| Wood frog | RsyD20 | 4 | PET | Julian and King 2003 |
