# Supplementary material for: Landscape genetics reveals unique and shared effects of urbanization for two sympatric pool‐breeding amphibians
Source: Ecol Evol. 2019 Oct 1;9(20):11799–823. doi: 10.1002/ece3.5685 (PMC6822048; doi:10.1002/ece3.5685)

Appendix 3. Density plots depicting values of environmental variables for each of the sampled vernal pools.


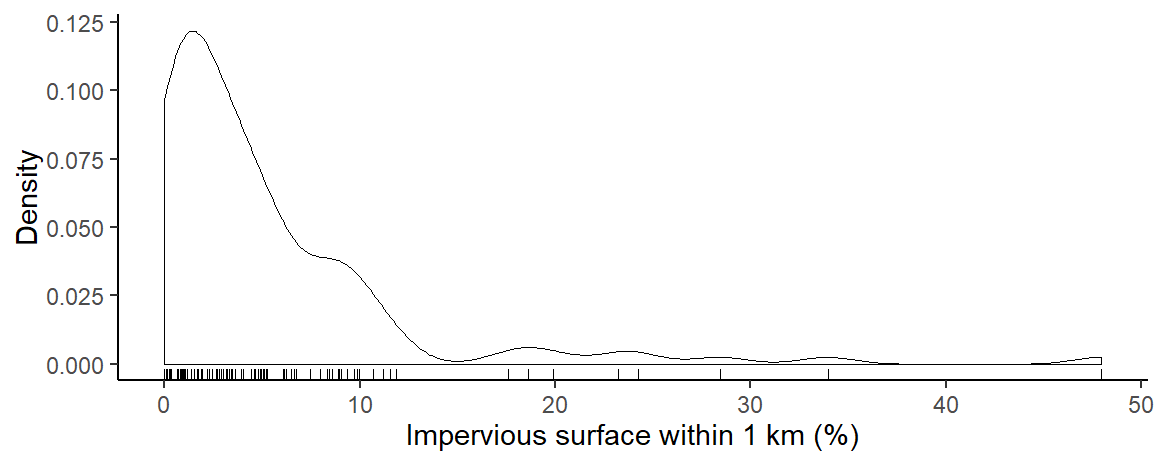


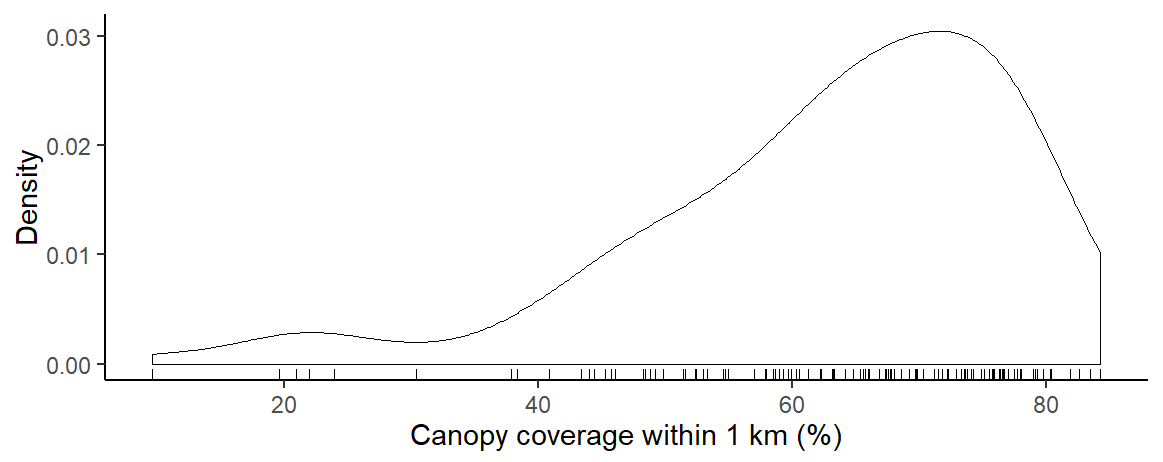


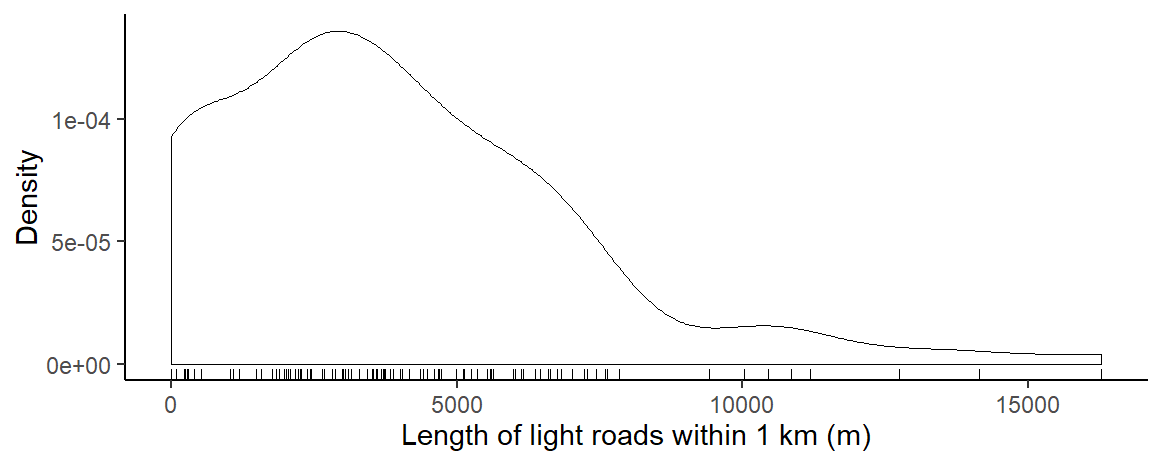


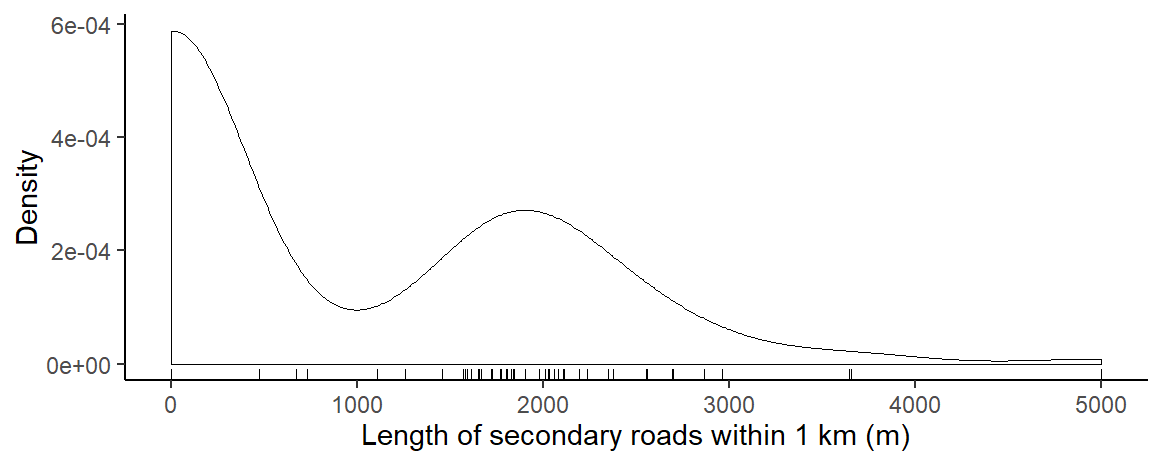

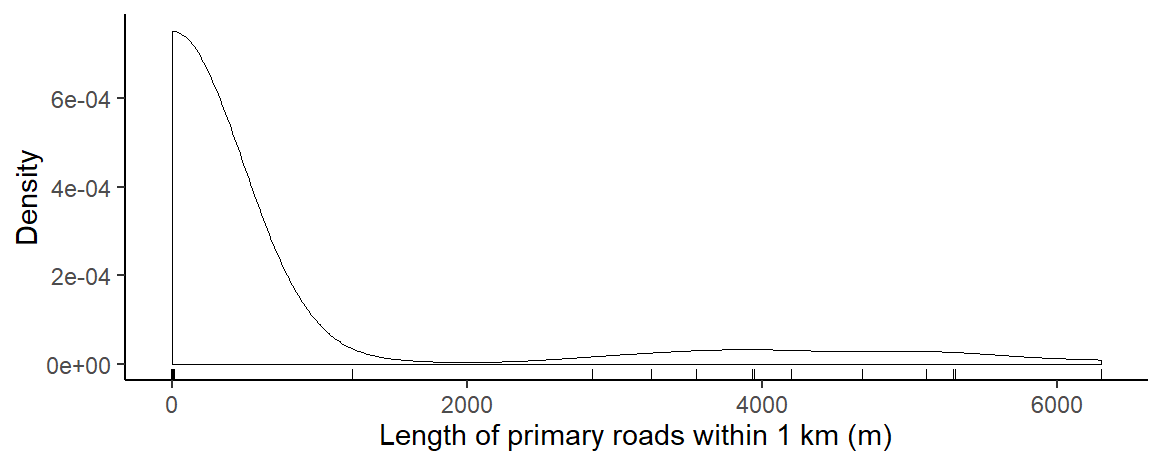

Supplement: Supplementary file 4 [file ECE3-9-11799-s004.docx]
